# Synaptosomes isolated from cryopreserved MND motor cortex reveal altered calcium handling and reduced complex IV–linked respiration

**DOI:** 10.3389/fnsyn.2026.1760254

**Published:** 2026-05-22

**Authors:** Sean D. Morrison, Lotten Ragnarsson, Sarah O. Cook, Joseph A. Rothnagel, Ernst Wolvetang, Bahaa Al-mhanawi, Mohammed R. Shaker, Thomas Robertson, Peter R. Dodd, Peter G. Noakes

**Affiliations:** 1 School of Biomedical Sciences, The University of Queensland, St Lucia, QLD, Australia; 2 Australian Institute for Bioengineering and Nanotechnology, The University of Queensland, St Lucia, QLD, Australia; 3 Institute for Molecular Bioscience, The University of Queensland, St Lucia, QLD, Australia; 4 School of Chemistry and Molecular Biosciences, The University of Queensland, St Lucia, QLD, Australia; 5 Neurological Disorders Research Center, Qatar Biomedical Research Institute, Hamad Bin Khalifa University, Qatar Foundation, Education City, Doha, Qatar; 6 Anatomical Pathology, Pathology Queensland, Brisbane, QLD, Australia; 7 Queensland Brain Institute, The University of Queensland, St Lucia, QLD, Australia

**Keywords:** complex IV, human post-mortem brain, mitochondria, motor cortex, motor neuron disease, neurodegeneration, respiration, synaptosomes

## Abstract

Motor neuron disease (MND) is marked by progressive neurodegeneration in which presynaptic Ca^2+^-handling and mitochondrial metabolism are thought to be vulnerable, but direct functional studies in human brain are scarce because most material is frozen long-term. Here, we show that synaptosomes isolated from paired fresh and experimentally frozen mouse cortex, and from cryopreserved human motor cortex, retain recognisable synaptosome ultrastructural features, synaptic proteome enrichment, and depolarisation-evoked Ca^2+^-mobilisation. K^+^ and veratridine elicited robust, pharmacologically suppressible Ca^2+^ influx across preparations, and response amplitudes in human samples varied by region but did not correlate with donor age, post-mortem interval (PMI), or years in storage. Synaptosomes from neuropathologically confirmed MND motor cortex and hSOD1^G93A^ mouse cortex showed significantly greater depolarisation-evoked Ca^2+^ entry than their respective controls, suggesting that increased presynaptic Ca^2+^ influx is shared across our human MND cohort and the hSOD1^G93A^ mouse model. Using synaptosome preparations from MND and control motor cortices in Seahorse respiratory assays, we found that Complex IV-driven oxygen consumption (TMPD/ascorbate–evoked and azide-sensitive) was reduced in MND synaptosomes, whereas donor-matched free-mitochondrial fractions showed no group difference, supporting a Complex IV defect detectable in the synaptosome-enriched fraction within this cohort. By defining protein-to-OCR relationships for both fractions, we provide practical parameters for applying these assays to archived human cohorts. Together, these data suggest that archived cryopreserved human brain tissues can support informative synaptosome Ca^2+^ and bioenergetic readouts, and that synaptosome-enriched preparations may reveal disease-relevant presynaptic phenotypes in MND that are not evident in donor-matched bulk mitochondrial isolates.

## Introduction

Synaptic terminals are among the most energy-demanding compartments in the nervous system. Neurotransmitter loading and release, rapid vesicle recycling, maintenance of ion gradients, and activity-dependent plasticity together impose a fluctuating ATP burden that is met by a combination of glycolysis and oxidative phosphorylation supplied by presynaptic mitochondria (Ly and Verstreken, 2006; Brand and Nicholls, 2011; Hafner et al., 2019; Faria-Pereira and Morais, 2022). At the same time, mitochondria tethered to presynaptic active zones must buffer large and rapid Ca^2+^ loads that arise from depolarisation and voltage-gated Ca^2+^ channel (VGCC) opening (Gerencser et al., 2009; Brand and Nicholls, 2011; Imai and Guarente, 2014; Mookerjee et al., 2017). This tight energetic–Ca^2+^ coupling makes synapses an early and sensitive site of failure in neurodegenerative conditions in which mitochondrial function, redox homeostasis, or axonal transport is compromised.

Motor neuron disease (MND), most commonly presenting as amyotrophic lateral sclerosis (ALS), is characterised clinically by progressive degeneration of upper motor neurons in motor cortex and lower motor neurons in brainstem and spinal cord (Robberecht and Philips, 2013). Converging evidence indicates that synaptic dysfunction and cortical hyperexcitability emerge early and may drive or accelerate downstream motor-system loss (Grosskreutz et al., 2010; Marinkovic et al., 2012; Chand et al., 2018; Hu et al., 2024). Multiple MND-linked pathways including RNA binding protein pathology, impaired axonal trafficking, glutamatergic dysregulation, Ca^2+^ mishandling, and mitochondrial stress, can converge at the presynaptic terminal, where modest defects in energy delivery or Ca^2+^ clearance may impair vesicle cycling and increase vulnerability to excitotoxic insults (Dupuis et al., 2004; Browne et al., 2006; Wang et al., 2016; Smith et al., 2019; Tefera et al., 2021; Yang, 2025). Yet, most of what is known about these mechanisms comes from transgenic mouse models or iPSC-derived neurons, which are extremely valuable but do not fully recapitulate the cellular composition, age, regional specialisation, and agonal variables present in end-stage human motor cortex (Studer et al., 2015; Justice and Dhillon, 2016). Direct functional interrogation of human synapses from neuropathologically confirmed MND brains remains an unresolved question.

Synaptosomes are resealed nerve-terminal particles generated during subcellular fractionation of cortical homogenates that retain presynaptic vesicles, mitochondria, and portions of the postsynaptic membrane. These preparations provide a tractable system to study human synaptic physiology *ex vivo* and, in parallel, yield a distinct free-mitochondrial fraction arising from disrupted cellular compartments (Whittaker et al., 1964; Dodd et al., 1986; Evans, 2015). Synaptosomes preserve stimulus-secretion coupling, support uptake and release assays, and, importantly, can be assayed for oxygen-consumption rate (OCR) and Ca^2+^ dynamics (Schenning et al., 2006; Choi et al., 2009; Choi et al., 2011). Synaptosomes can be prepared from previously cryopreserved human or rodent brain, although uptake (e.g., dopamine) is often reduced compared with fresh preparations, and their homogeneity and viability have not been systematically evaluated (Eshleman et al., 2001; Dunkley et al., 2008). Nevertheless, post-mortem studies in epilepsy and schizophrenia have shown that cryopreserved human cortex can yield synaptosomes suitable for transmitter-release and receptor-binding assays (Dodd et al., 1981; Nagy and Delgado-Escueta, 1984; Dodd et al., 1986; Hardy et al., 1986; Sherman et al., 1991). Functional studies on synaptosomes prepared from biobanked cryopreserved human cortex have remained limited and have rarely been benchmarked against fresh material.

Updated bioenergetic protocols have enabled profiling of electron transport chain (ETC) complex-specific mitochondrial function from frozen tissues (Acin-Perez et al., 2020). These functional synaptosome endpoints provide a practical framework for interrogating archived human control and neuropathologically confirmed MND cortices. By comparing synaptosome fractions with free-mitochondrial fractions, the latter representing the mixed population of mitochondria isolated from tissue homogenates (i.e., non-synaptosome mitochondria), from the same human donor samples, it becomes possible to determine whether bioenergetic deficits are restricted to synapses or reflect broader mitochondrial impairment in MND.

Here, we address this unresolved question by testing whether long-term cryopreserved human cortex can still yield functional synaptosomes. We show that these preparations retain (i) recognizable synaptosome ultrastructure and synaptic proteome; (ii) depolarisation-evoked Ca^2+^ influx via Na_V_/VGCC-dependent pathways; and (iii) sufficient respiratory-chain integrity to resolve Complex IV-specific OCR responses. Ca^2+^ entry was stimulus-dependent (KCl, veratridine), partially sensitive to broad and subtype-selective Ca^2+^ channel blockade and varied across cortical/subcortical regions independently of age, PMI, or years in storage. Importantly, synaptosomes derived from human MND motor cortex and from hSOD1^G93A^ mice showed greater depolarisation-evoked Ca^2+^ influx than their respective controls, suggesting that increased Ca^2+^ entry is shared across the human MND cohort studied here and the hSOD1^G93A^ mouse model. We also asked whether this material preserves functional mitochondrial readouts. Synaptosomes from MND motor cortex showed a selective reduction in TMPD/Ascorbate–driven Complex IV respiration, whereas donor-matched free-mitochondrial fractions did not, suggesting that the observed defect is detectable in the synaptosome-enriched fraction. Defining protein–OCR scaling in both fractions clarifies how cryopreserved human material can be used for future screening and multi-omic integration. Together, these data show that cryopreserved human CNS tissue can support synaptic functional assays, providing a practical platform for detecting presynaptic phenotypes in MND and potentially in other disorders with altered mitochondrial function.

## Materials and methods

### Human brain collection and ethical considerations

Unfixed, cryopreserved tissue samples were obtained from the Queensland Brain Bank (QBB), School of Chemistry and Molecular Biosciences located at the University of Queensland, in accordance with the National Health and Medical Research Council’s national statement on ethical conduct on human research (2007; updated 2018; Australian National Health and Medical Research Council, the Australian Research Council and Universities Australia, Commonwealth of Australia, Canberra). Further, this study was conducted in accordance with the Declaration of Helsinki Principles (Bulletin of the World Health Organization 2001). Research using human tissue proceeded with informed written consent from the next of kin, and histopathological examination performed by neuropathologists at the Royal Brisbane and Women’s Hospital Queensland, to confirm neurological diagnoses. Histopathological examination of MND-affected donors consistently demonstrated upper and lower motor neuron degeneration, with neuronal loss and gliosis in the motor cortex, cranial nerve nuclei, and spinal anterior horn cells. Pyramidal tract degeneration and sparse, non-specific ubiquitin-positive inclusions were variably observed, while tau and *β*-amyloid pathology was minimal. These findings support a primary diagnosis of motor neuron disease and confirm that all cases met the criteria for classification as affected or unaffected. Clinical diagnosis, disease duration and neuropathological findings in post-mortem MND cases are presented in Supplementary Table S1. Patients were de-identified, and their case information including age, post-mortem interval and clinical diagnosis are reported in Table 1. Where possible, the PMI between death and autopsy removal of the brain was less than 48 h; neural proteins remain stable within this period (Blair et al., 2016). All procedures had been approved by the Human Ethics Research Committees of RWBH (ref. 2008/040), The Prince Charles Hospital (ref. HREC/10/QPCH/63) and The University of Queensland (ref. 2009001255).

**Table 1:** Human post-mortem donor and specimen information, including assay allocation, sampled region, sex, age, post-mortem interval (PMI), years in storage (YIS), and diagnosis.

Study	Region used	Sex	Age (yr)	PMI (hr)	YIS	Diagnosis
FLIPR	Inferior frontal/occipital	F	76	24	26.46	Control
FLIPR	Superior temporal	F	57	9.7	21.05	Control
Seahorse	Posterior motor	F	77.84	59	20.17	Control
Seahorse	Posterior motor	F	68.33	8	17.55	Control
FLIPR	Mid frontal	F	75	48.75	16.55	Control
FLIPR	Mid caudate	F	87	27.05	16.15	Control
FLIPR, TEM	Anterior/posterior motor/occipital	M	67	29	13.65	Control
Seahorse, TEM	Posterior motor	M	87.25	78	13.71	Control
FLIPR	Mid caudate/mid temporal/occipital	F	77	17.83	12.37	Control
FLIPR	Mid frontal	M	80	47.25	12.05	Control
FLIPR	Pallidus putamen	M	76	25.5	9.2	Control
*Control Mean ± SD*			*74 ± 8.7*	*34 ± 22*	*17 ± 3.2*	
Seahorse	Posterior motor/occipital	M	84.76	35	18.93	MND^1^
FLIPR	Posterior motor	M	52	24.75	12.86	MND^2^
FLIPR	Posterior motor	F	57	22.25	11.8	MND^3^
Seahorse	Posterior Motor/occipital	M	65.24	59.3	12.39	MND^4^
Seahorse	Posterior Motor/occipital	M	53.75	44.58	12.36	MND^5^
FLIPR, Seahorse	Anterior motor/posterior motor	M	51	22.25	11.14	MND^6^
Seahorse	Posterior motor	M	87.62	31.5	11.53	MND^7^
*MND mean ± SD*			*62.8 ± 15*	*32.7 ± 13*	*13.8 ± 3.4*	

MND1-7 reflects individual unique cases used.

### Mouse tissue and ethical considerations

Age-matched male mouse cortices from C57BL/6 [strain control (wild-type)] and male B6. Cg-Tg(SOD1^G93A^)1Gur/J, carrying a high-copy human SOD1^G93A^ transgene, a well-established familial ALS model (Gurney et al., 1994) were obtained by the Queensland Brain Institute animal facility. Male mice were used exclusively, as previous studies report earlier disease onset and more rapid progression in hSOD1^G93A^ males compared to age-matched females (Gurney et al., 1994; Veldink et al., 2003; Heiman-Patterson et al., 2005). This research was approved by The University of Queensland Animal Care and Ethics Committee and conducted in accordance with the Queensland Government Animal Research Act 2000, associated Animal Care and Protection Regulation (2002 and 2008), and the Australian Code for the Care and Use of Animals for Scientific Purposes (National Health and Medical Research Council, 2013). Work was conducted under The University of Queensland Animal Ethics Committee Approval No. 398/18/NHMRC.

### Synaptosome preparation

Mouse cortices (~400 mg) were collected following euthanasia via cervical dislocation and kept at 4 °C in sucrose buffer (0.32 M sucrose, 20 mM HEPES, pH 7.4) prior to subcellular fractionation of synaptosomes. For benchmarking, paired fresh and frozen mouse cortical samples were prepared. Frozen mouse tissue was generated by sagittal hemi-section of cortices, followed by slow freezing in iso-osmotic cryoprotectant using a protocol adapted from Dodd et al. (1986). These samples were stored at −80 °C for a minimum of 2 months prior to processing and we distinguish as ‘frozen mouse’, whereas ‘fresh mouse’ tissue was processed immediately following dissection. Human cortical tissue (~500 mg per sample) was obtained from the QBB following post-mortem autopsy and neuropathological assessment; these tissues had been stored at −80 °C for extended periods (~9–26 years). These archived samples are referred to here as cryopreserved tissue. Samples were stored in individual polyethylene bags labelled by case and anatomical region, rapidly thawed in 37 °C sucrose buffer (Dodd et al., 1981), weighed, and maintained at 4 °C in sucrose buffer prior to subcellular fractionation.

Synaptosomes from these human and mouse brain samples were then prepared as described previously (Dodd et al., 1981; Dodd et al., 1986; Chang et al., 2015). In brief, human and mouse brain tissue samples were homogenised with 8 downward strokes using a motor-driven Teflon–glass (Potter–Elvehjem) homogeniser (WHEATON; 15 mL) equipped with a loose-fitting PTFE pestle (~0.15 mm clearance) with 12 mL of 0.32 M (4 °C) sucrose containing a phosphatase inhibitor cocktail (1 mM Na^+^ Orthovanadate, 10 mM Na^+^ Pyrophosphate, 2 mM *β*-Glycerol Phosphate and 30 mM Na^+^ fluoride). The homogenate was centrifuged at 5000 g for 10 min at 4 °C in a Beckman Coulter OptimaTM XPN-100 ultracentrifuge. The P1 pellet contained debris and nuclear fragments and was discarded. The S1 (low-speed supernatant) underwent successive density-gradient centrifugation steps in the SW41Ti swing-out rotor to enrich the mitochondrial and synaptosomal fractions. S1 was layered onto 4 mL of 1.2 M sucrose buffer and centrifuged at 40,000 g for 16 min at 4 °C. The resultant P2 pellet enriched in mitochondria was used as a biological control for subsequent respiration studies; it was stored at 4 °C for a minimum 2 h and resuspended in mitochondrial assay solution (see below). The 1.2 M sucrose interface layer was carefully isolated and diluted with 0.32 M sucrose buffer, then layered onto 4 mL of 0.8 M sucrose buffer. The second gradient was centrifuged at 40,000 g for 16 min at 4 °C. The pellet (P3) enriched in synaptosomes was resuspended in synaptosome assay solution and stored at 4 °C for a maximum of 2 h before respiratory measurements were performed. These steps are summarised in Supplementary Figure S1.

### Fluorometric calcium-4 recordings of synaptosomes

Synaptosome samples were diluted to 8 μg/μl protein in physiological saline solution (PSS; 140 mM NaCl, 11.5 mM glucose, 5.9 mM KCl, 1.4 mM MgCl_2_, 1.2 mM NaH_2_PO_4_, 5 mM NaHCO_3_, 1.8 mM CaCl_2_, 10 mM HEPES, pH 7.4; adjusted with NaOH). Protein measurements were made by Nanodrop One (Thermo Fisher Scientific, USA) using A280/280 settings. Synaptosomes (17 μL) were seeded into black-walled, CellBIND® 384-well Clear Bottom Microplates (Corning, MA, USA) and briefly centrifuged at 3500 rpm for 10 min to attach synaptosomes to the bottom of the plate. All processes remained at 4 °C before incubation and Ca^2+^ measurements. Synaptosomes were loaded with 3 μL (of 10x) Calcium-4 No-wash dye (Molecular Devices, CA, USA) by diluting the lyophilized dye in PSS and were incubated for 10 min at 37 °C in a 5% humidified CO_2_ incubator. Incubation time of 10 min was found to yield the optimal fluorescent signal. Intracellular relative fluorescence units (RFU) in response to potassium chloride (KCl) was measured in a Fluorometric Imaging Plate Reader (FLIPR™; Molecular Devices) using a cooled CCD camera with excitation at 470–495 nm and emission at 515–575 nm (Vetter, 2012). Camera gain and intensity were adjusted for each plate to achieve a baseline fluorescence of at least 1,000 arbitrary fluorescence units. For pharmacological inhibition of Ca^2+^ measurements, synaptosomes were preincubated with Tetrodotoxin (TTX; 1 μM), *ω*-Agatoxin-IVA (2 μM), ω-Conotoxin-CVID (1 μM), and Nifedipine (10 μM) (Alamone Labs, Israel) for 10 min prior to ligand stimulation. For TTX pre-treatment experiments, synaptosomes were stimulated with veratridine (50 μM) (Sigma-Aldrich, USA). Prior to addition of agonist, 10 baseline fluorescence readings were taken, followed by fluorescent readings every second for ~350 s. During operation of the FLIPR machine, 10 μL of the ligand (in PSS) was added to the appropriate well, denoted by a stimulation arrow. Mean traces for each condition (± SEM) were generated following spatial uniformity correction, with baseline fluorescence normalised to 100. Changes in fluorescence were expressed as ΔF/F₀ (% of baseline), where F₀ was defined as the minimum fluorescence value across the initial baseline measurements and F as the peak fluorescence during the activation phase (first 60 s following stimulation). Area under the curve (AUC) was calculated from ΔF/F₀ traces where indicated.

### Seahorse respirometry analysis of synaptosomes and mitochondria from cryopreserved brain tissues

Synaptosome and mitochondrial oxygen consumption rates (OCR) were monitored in the Seahorse XFe96 Analyser (Seahorse Bioscience, Agilent Technologies, CA, USA). Synaptosomes prepared on the day of the assay were resuspended in a modified synaptosome assay solution (SAS), supplemented with substrates for oxidative phosphorylation as previously described [3.5 mM KCl, 120 mM NaCl, 1.3 mM CaCl_2_, 0.4 mM KH_2_PO_4_, 1.2 mM Na_2_SO_4_, 2 mM MgSO_4_, 15 mM d-Glucose and 10 mM Na^+^-Pyruvate, pH 7.2; (Choi et al., 2009, 2011)]. Mitochondria prepared during synaptosome enrichment were resuspended in mitochondrial assay solution (MAS) as described previously [70 mM sucrose, 220 mM mannitol, 10 mM KH_2_PO_4_, 5 mM MgCl_2_, 2 mM HEPES, 1 mM EGTA and 0.2% (w/v) fatty acid-free BSA, pH 7.2 (Rogers et al., 2011)]. Each biological sample (30 μL) was seeded into a Seahorse XFe96 microwell plate, previously coated overnight with Polyethylenimine [PEI; 66.7 ng/mL (1:15,000)] to promote attachment (Aktories et al., 2022). The XFe96 plate was centrifuged at 2500 g for 30 min at 4 °C. A total of 150 μL SAS or MAS was then gently added, supplemented with cytochrome c (10 μg/mL; C2506, Sigma-Aldrich, MO, USA) and alamethicin (1 μg/mL; A4665, Sigma-Aldrich). Alamethicin was used to permeabilise membranes to NADH while preserving respiratory enzyme function (Gostimskaya et al., 2002). The hydrated sensor cartridge was calibrated in the system as per the manufacturer’s instructions for initialization. For synaptosomes and mitochondria, the injection ports were subsequently loaded with the following: 1 mM NADH (port A); 2 μM Rotenone and Antimycin A (Rot/AA; port B); 1 mM N, N, N′, N′-Tetramethyl-p-Phenylenediamine (TMPD) and 0.5 mM Ascorbic acid (port C); and 50 mM Na^+^ Azide (port D). The microplate was incubated at 37 °C for approximately 15 min before loading into the Seahorse analyser for oxygen and pH equilibration. Experimental measurements were conducted at 37 °C. Technical replicates of 5–7 were used for each biological sample. All solutions were prepared using high-purity deionized water, and the pH of SAS and MAS solutions were adjusted to 7.2 using potassium hydroxide (KOH) or hydrochloric acid (HCl) when required. All reagents and solutions were prepared fresh on the day of the assays including cytochrome c and alamethicin.

### Seahorse respirometry normalization and data processing

All OCR traces were plotted as OCR pmol O₂/min, normalised to protein loading (per μg protein, per well) and represented as the mean ± SEM. When synaptosomes and mitochondria were loaded in the same plate, background wells for both SAS and MAS were included and assigned to a new ‘background’ variable in the Wave Bioscience program (version 2.6; Agilent Technologies) for retrospective subtraction of sample-negative wells. Auto-oxidation controls for MAS and SAS were inspected post-hoc to ensure wells without biological sample were true background (Supplementary Figure S2A). Permeabilised and unpermeabilised controls were included; NADH addition increased OCR in permeabilised conditions only (Supplementary Figure S2B). Complex IV activity was quantified as OCR (pmol O₂/min per μg protein) following TMPD/Ascorbate stimulation (maximal respiration) subtracted by lowest Azide-sensitive respiratory measurement, to account for non-mitochondrial oxygen consumption. Following the conclusion of the OCR assay, synaptosome and mitochondrial samples were centrifuged at 2500 g for 30 min at 4 °C, washed and loaded with MitoTracker™ 647 nm (Thermo Fisher Scientific), final concentration 250 nM, for 30 min at 37 °C. MitoTracker measurements were acquired immediately after the assay and included unstimulated controls. XFe96 assay plates were subsequently washed and loaded into the SPECTROstar Nano (BMG Labtech Pty. Ltd., VIC, Australia). The protocol was set up to input compatible Seahorse XFe96 well microplates for analysis: LxWxH; 127.76, 85.47, 17.4, X/Y offset from A1:14.38, 11.24. Base, Depth, Stack. Absorbance in the 647 nm wavelength was used for mitochondrial event normalization. Protein measurements were subsequently made with the PierceTM BCA Protein Assay Kit (ThermoFisher scientific) as per the manufacturer’s instructions and measured at 562 nm.

### Western blot

Fractions were lysed using Pierce™ RIPA Buffer (ThermoFisher Scientific, Cat. #89900) supplemented with Halt Protease & Phosphatase Inhibitor Cocktail (ThermoFisher Scientific, Cat. #1861281), with a final volume ratio of 1:4; fraction: lysis buffer. Protein concentration of the lysate was determined using the Pierce™ BCA protein assay kit (ThermoFisher Scientific, Cat. #23227) as per the manufacturer’s instructions. Samples were heated at 100 °C for 10 min before loading. Equal amounts of protein were loaded onto Mini-PROTEAN TGX Stain-Free Gels (Bio-Rad, Cat. #4568044), followed by electrophoresis (100 V, 45 min) and transferred onto iBlot 2 PVDF Mini Stacks (Invitrogen, Cat. #IB24002). The membrane was blocked for 1 h at room temperature with 5% skim milk in TBST (20 mM Tris–HCl, pH 7.6, 136 mM NaCl, 0.1% Tween-20). Primary antibodies, including rabbit-anti-Synapsin-1 (D12G5, 5297; Cell Signalling; 1:1000), mouse-anti-TOM20 (MABT166; Millipore; 1:1000) and loading control mouse-anti-beta-Actin (8H10D10, 3700S; Cell Signalling; 1:1000), were incubated with the membrane in 5% BSA in 1 x TBST for 12 h at 4 °C. The membrane was then washed three times with 1 × TBST for 10 min each at room temperature, followed by incubation with HRP-conjugated secondary antibodies diluted 1:5000 in 5% skim milk in 1 × TBST for 1 h at room temperature. Final washes were performed three times with 1 × TBST for 10 min each at room temperature, and visualization was completed using Clarity Western ECL Substrate (Bio-Rad, Cat. #170–5060). Band intensities were quantified as integrated density and normalised to the corresponding loading control. Raw, uncropped blots are shown in extended data.xlsx, Figure 5 tab.

### Statistical analyses and data presentation

Data were assessed for distributional assumptions where sample size permitted; however, normality testing was interpreted cautiously for comparisons involving small donor cohorts. Data were analysed using GraphPad Prism (versions 8.0 and 9.0). Technical replicates were averaged within each biological sample (e.g., donor, mouse, or independent preparation), and statistical comparisons were performed at the biological replicate level. Two-group comparisons were assessed using unpaired two-tailed *t*-tests with Welch’s correction. Where appropriate, multiple-group comparisons were performed using one-way or two-way ANOVA with multiple-comparisons testing, as specified in the figure legends. Data are presented as mean ± SEM unless otherwise indicated. For distributional visualisation, violin and box plots display the full data distribution with median and interquartile range. *p* values ≤ 0.05 were considered statistically significant (*p ≤ 0.05, **p ≤ 0.01, ***p ≤ 0.001, ****p ≤ 0.0001). Final figures were assembled using Adobe Illustrator (Version 21: Adobe Inc. 2019).

## Results

### Human cases

Histopathological examination of brain tissue was performed by the Pathology Queensland Neuropathology section at the Royal Brisbane and Women’s Hospital. Donor demographic and neuropathological information is summarised in Table 1. Control material comprised 11 tissue specimens obtained from neuropathologically normal donors (7 female, 4 male), spanning multiple cortical and subcortical regions. These specimens were allocated across functional assays, including FLIPR Ca^2+^ measurements (*n* = 8), Seahorse respirometry (*n* = 4), and transmission electron microscopy (TEM; *n* = 3), with some samples used across multiple assays (Table 1). MND material comprised tissue from seven donors (6 male, 1 female), including motor cortices. In total, 8 tissue specimens were analysed, with allocation to FLIPR Ca^2+^ assays (*n* = 3) and Seahorse respirometry (*n* = 5). Occipital cortex, a region relatively spared in MND, was included as an internal regional control (Brettschneider et al., 2013; Walhout et al., 2015; Machts et al., 2018). Donor age ranged from 57 to 87 years in controls (mean ± SD: 74 ± 8.7) and 51–87 years in MND cases (62.8 ± 15). Post-mortem interval (PMI) ranged from 9.7 to 78 h in controls (34 ± 22) and 22.3–59.3 h in MND cases (32.7 ± 13). Years in storage (YIS) ranged from 9.2–26.5 years in controls (17 ± 3.2) and 11.1–18.9 years in MND cases (13.8 ± 3.4). All tissue specimens were processed to isolate synaptosome-enriched fractions; subsets were used for free-mitochondrial isolation for Seahorse assays, with allocation detailed in Table 1.

### Synaptosomes isolated from cryopreserved human tissue show synaptosome ultrastructural features comparable to fresh and frozen mouse brain preparations

**Figure 1:**
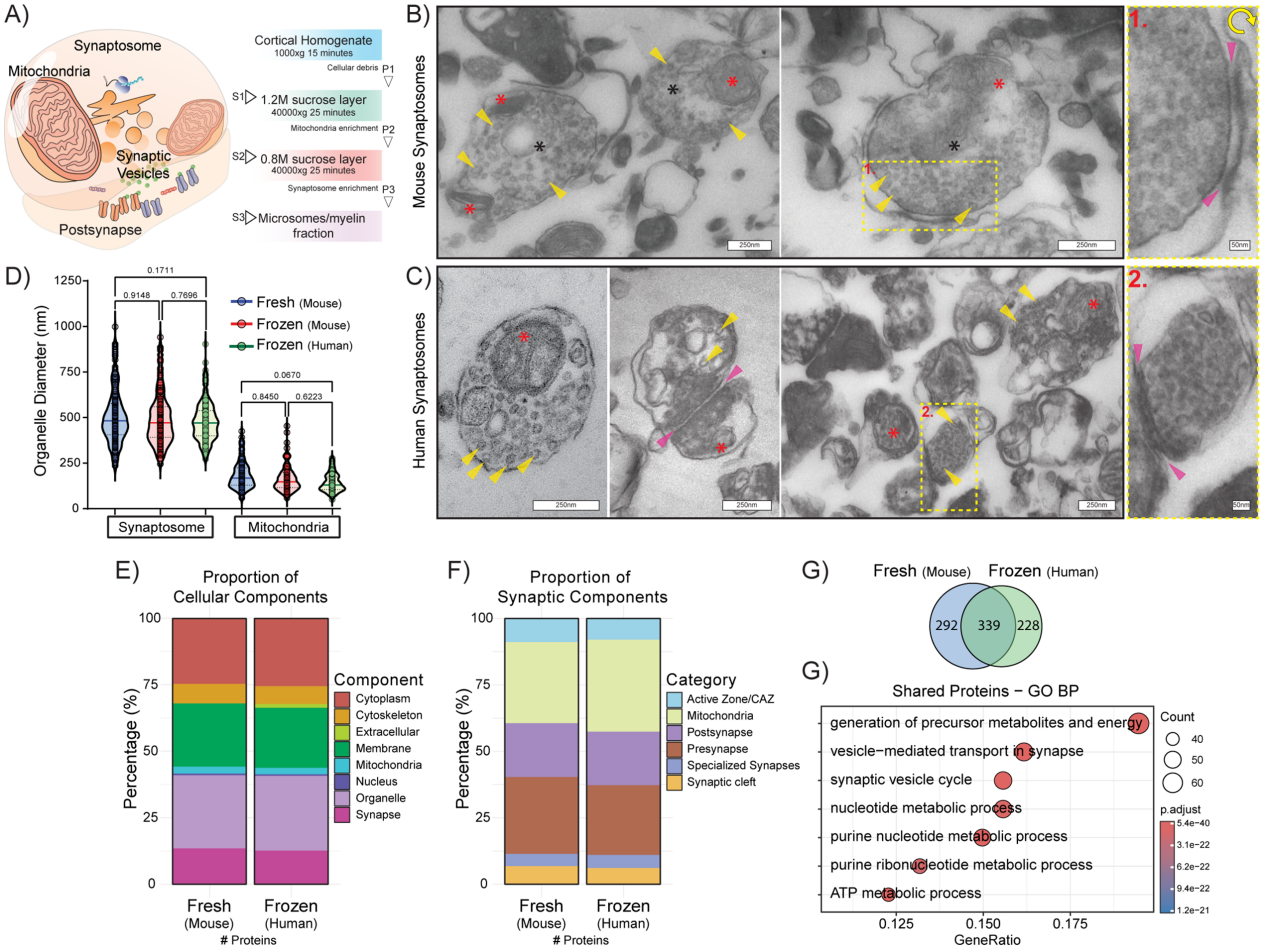
Synaptosomes isolated from fresh and frozen mouse cortex as well as cryopreserved human cortical tissue preserve synaptosome-like morphology and synaptic proteomic enrichment. **(A)** Representative schematic of synaptosome with mitochondria, post-synaptic density, and synaptic vesicles. Graphical illustration (right) of density-gradient centrifugation synaptosome isolation with 0.32 M sucrose; see Supplementary Figure S1. **(B,C)** Representative electron micrographs of synaptosome-enriched fractions from fresh mouse cortex **(B)** and cryopreserved human cortex **(C)**. Synaptic vesicles (20–40 nm in diameter) are visible in yellow highlighted insets. Scale bars = 250 nm, Inset Scale bar = 50 nm. Black asterisks denote synaptosomes, red asterisks denote mitochondria, yellow arrows denote synaptic vesicles, and pink arrows denote synaptic clefts. **(D)** Violin plots of synaptosome (left) and mitochondria (right) organelle diameter from synaptosome fractions isolated from freshly prepared mouse cortex (blue), frozen mouse cortex (red) and cryopreserved human autopsy material (green). **(E,F)** Proportion of top-level *Gene Ontology* (GO) cellular component categories and synaptic component categories in fresh and cryopreserved synaptosome proteomes. The stacked bar graphs show the relative distribution (%) of proteins assigned to each broad cellular component category **(E)** and each defined synaptic component category **(F)** within each proteome (*n* = 1 per proteome). **(G)** Venn diagram showing the overlap of protein-coding genes between fresh mouse (blue) and cryopreserved control human (green) synaptosome proteomes; numbers indicate shared and unique genes. Protein sets were derived from UniProt accession annotations. **(G’)** Top enriched GO biological processes (BP) among shared proteins. Dot plot showing the top 7 significantly enriched BP terms; dot position reflects gene ratio, size indicates gene count, and colour denotes adjusted *p*-value significance (*n* = 1 per proteome).

The homogeneity and viability of synaptosomes prepared from frozen brain tissue has not been thoroughly compared to freshly prepared tissue (Eshleman et al., 2001; Dunkley et al., 2008). To address this issue, we first performed paired isotonic sucrose cryopreservation of brain tissue from mouse and human autopsy material (Figure 1A). Synaptosomes prepared from fresh, frozen mouse tissue and cryopreserved human cortical tissue all showed characteristic synaptosome morphologies determined by transmission electron microscopy (TEM; Figures 1B,C). We note the freshly prepared mouse- and cryopreserved human-derived synaptosomes contained synaptic vesicles (yellow arrows) and adjacent electron-dense structures consistent with postsynaptic densities (dual pink arrows; Figures 1B,C). Mitochondria enclosed within synaptosome-like structures could be identified, although assessment of cristae was limited given the permeabilization of mitochondrial membranes following freeze–thaw (asterisks in Figure 1C); careful examination of these structures is also presented in Supplementary Figure S3. No significant changes were observed in synaptosome diameter or inter-synaptosome mitochondrial diameter between fresh or frozen mouse and cryopreserved human preparations (Figure 1D).

To complement our ultrastructural assessments of synaptosome preparations, we performed mass spectrometry–based proteomic analysis to compare the molecular composition of fresh and cryopreserved samples. Synaptosomes derived from fresh mouse- and cryopreserved human brain were enriched for *Synapse* and *Organelle*-related Gene Ontology (GO) cellular component terms, with minimal representation of nuclear components (Figure 1E). Analysis of synaptic component categories indicated the presence of both pre- and postsynaptic elements, including active zone and synaptic cleft proteins, in both fresh-mouse and cryopreserved human synaptosome preparations (Figure 1F). Shared proteins between fresh-mouse and cryopreserved human synaptosomes were enriched for *biological processes* such as *vesicle-mediated transport in synapse*, *synaptic vesicle cycle* and various metabolic processes, which together support the preservation of core synaptic functions in synaptosomes isolated following freeze–thaw of cryopreserved human brain tissue (Figure 1G). Collectively, these data support that synaptosome-enriched fractions isolated from cryopreserved human brain retain molecular features comparable to those derived from fresh mouse tissue.

### Retention of depolarisation-evoked Ca^2+^ signalling in synaptosomes from cryopreserved human cortex

**Figure 2:**
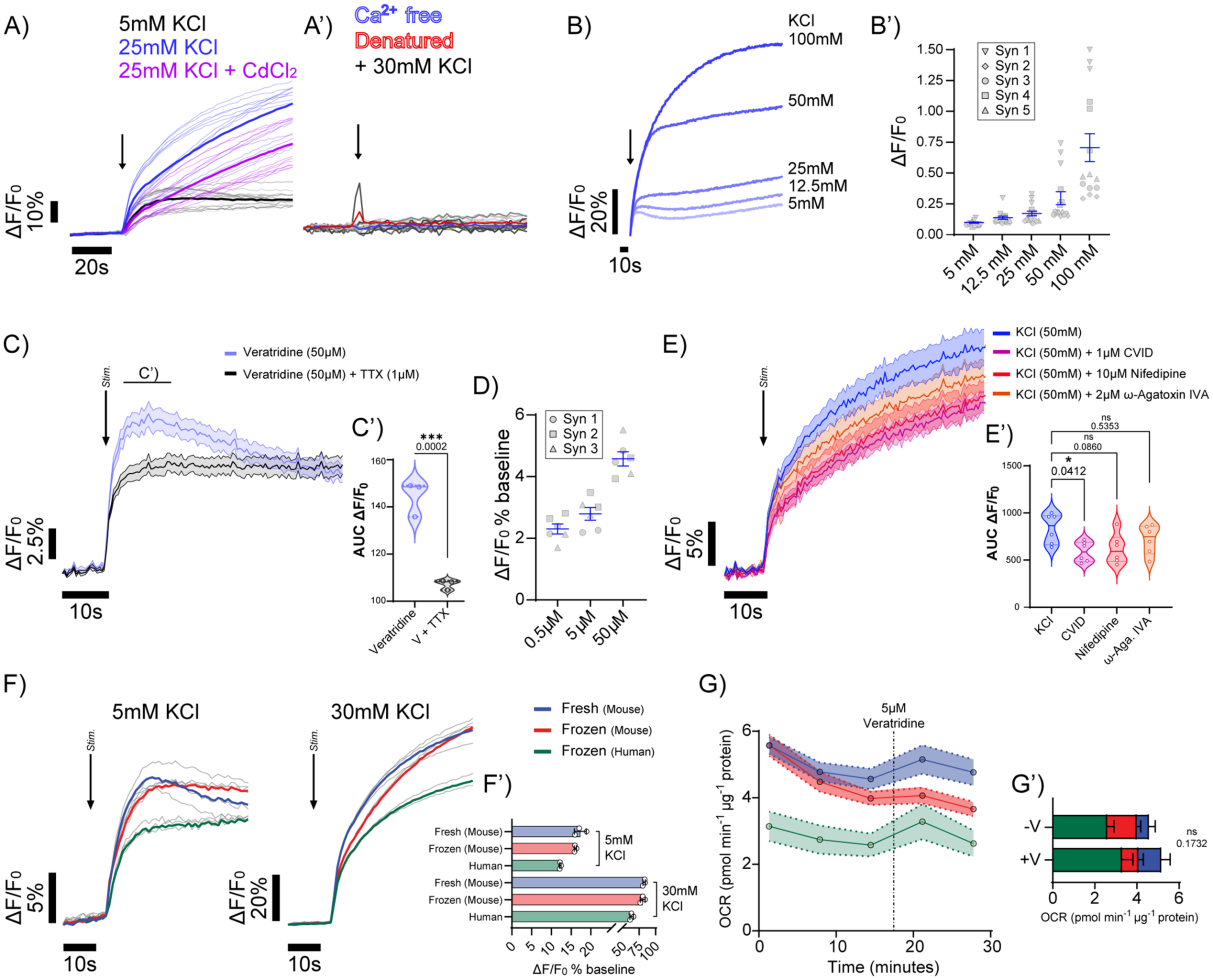
Validation of cryopreserved tissue derived synaptosomes for functional assays of depolarization and Ca^2+^ influx. **(A)** Representative Ca^2+^ influx traces in response to 25 mM KCl (blue) and KCl + CdCl_2_ (purple), showing reproducible depolarisation-induced responses across technical replicates. Solid line represents the mean; opaque lines represent replicates. **(A’)** Control traces demonstrating abolished Ca^2+^ influx in Ca^2+^-free buffer (blue) and denatured synaptosomes (red), validating specificity of the signal. **(B)** Graded Ca^2+^ influx responses to increasing KCl concentrations (5–100 mM, blue) demonstrating concentration-dependent depolarization of the synaptic membrane. **(B′)** Quantification of peak Ca^2+^ mobilization across KCl concentrations, shown as individual replicates (symbols) with mean ± SEM overlay; *n* = 5 independent preparations. **(C)** Depolarization with 50 μM veratridine (violet-blue) compared to TTX-pretreated synaptosomes (black), highlighting robust FLIPR Ca^2+^ signals; mean trace ± SEM (*n* = 3 biological replicates). **(C′)** Violin plots of normalised area-under-the-curve (AUC) responses showing significant increases with 50 μM veratridine versus TTX-pretreated for the initial 10 s stimulation; *n* = 3 preparations. **(D)** Quantification of peak Ca^2+^ mobilization across veratridine concentrations, shown as individual replicates (symbols) with mean ± SEM overlay; *n* = 3 independent preparations. **(E)** Ca^2+^ mobilization in response to 50 mM KCl following pre-incubation with Ca^2+^ channel blockers: *ω*-Agatoxin IVA (P/Q-type, 2 μM; orange), Nifedipine (L-type, 10 μM; red), ω-Conotoxin CVID (N-type, 1 μM; purple). **(E’)** AUC analysis of blocker experiments (60 s), demonstrating reduced total Ca^2+^ mobilization by all blockers, with significant suppression by ω-Conotoxin CVID (*p* < 0.05); *n* = 3 independent preparations. **(F)** Comparative overlays of Ca^2+^ influx in synaptosomes prepared from fresh mouse (blue), frozen mouse (red), and cryopreserved human (green) brain tissue in response to 30 mM KCl. **(F′)** Quantification of Ca^2+^ influx across fresh mouse, frozen mouse, and cryopreserved human synaptosomes (AUC normalized to synaptosome protein, μg). Calcium mobilization is lower in human synaptosomes (****p < 0.0001), with no significant difference between fresh and frozen mouse synaptosomes (*p* = 0.054); *n* = 3 per group. **(G)** Oxygen consumption rate (OCR) traces of synaptosomes from fresh mouse (blue), frozen mouse (red), and cryopreserved human (green) brain tissue under basal conditions and following veratridine stimulation (3 μM, dotted line). Mean trace ± SEM, *n* = 3 biological replicates, 4 technical replicates. **(G’)** Quantification of basal and stimulus-induced OCR across groups. Cryopreserved human synaptosomes show reduced basal respiration (*p* = 0.011), but the change after stimulation was not significantly different under these assay conditions (*p* = 0.58).

To functionally assess the membrane integrity of synaptosomes prepared from cryopreserved tissues, Ca^2+^ dynamics were assessed following KCl-depolarisation. Addition of 25 mM KCl to human synaptosomes increased ΔF/F₀, and this response was attenuated by pre-incubation with the broad-spectrum high-voltage–activated Ca^2+^ channel blocker CdCl_2_ (Figure 2A). Ca^2+^ influx was abolished following KCl depolarisation without extracellular Ca^2+^ present and following synaptosomal denaturation (Figure 2A’), supporting the interpretation that the observed Ca^2+^ effects described above depend on intact synaptosome structure. Increasing KCl concentrations (5–100 mM) produced a concentration-dependent increase in ΔF/F₀ in human synaptosomes, which was consistent across independent preparations (Figures 2B,B’). To distinguish the Ca^2+^ entry driven by global KCl-induced depolarisation, from channel-mediated activation, we used veratridine (50 μM) to engage voltage-gated Na^+^ channels. Veratridine treatment produced a robust rise in ΔF/F₀ that was significantly greater than the response observed with veratridine in the presence of tetrodotoxin (TTX; 1 μM) during the first 15 s (Figures 2C,C’). Although TTX pretreatment largely suppressed Na^+^ channel-dependent depolarisation, a residual Ca^2+^ elevation persisted, consistent with non-TTX-sensitive mechanisms such as Na^+^/Ca^2+^ exchange or intracellular store release. Veratridine-evoked Ca^2+^ influx increased in a concentration-dependent manner (Figure 2D). To determine the Ca^2+^ entry pathways underlying synaptosomal depolarization, we examined the effect of selective voltage-gated calcium channel (VGCC) blockade on Ca^2+^ mobilisation induced by 50 mM KCl. Pre-incubation with the P/Q-type channel blocker *ω*-agatoxin IVA (2 μM), the N-type blocker ω-conotoxin CVID (1 μM), or the L-type blocker nifedipine (10 μM) each produced modest attenuation of the KCl-evoked ΔF/F₀ compared to KCl alone (Figures 2E,E’).

We next assessed depolarization-induced Ca^2+^ influx at 5 mM and 30 mM KCl in synaptosomes derived from fresh and frozen mouse brain and cryopreserved human brain, benchmarking responses against fresh tissue (Figure 2F). 5 mM KCl elicited small ΔF/F₀ increases, whereas 30 mM KCl produced larger responses. Frozen mouse synaptosomes were comparable to fresh, while synaptosomes from human tissues showed reduced amplitudes (Figure 2F’). To determine whether Naᵥ-dependent depolarisation engages mitochondrial respiration, we measured OCR in synaptosome preparations following veratridine (5 μM) stimulation. Veratridine produced a modest increase in OCR across samples; however, this effect did not reach significance after normalisation, and endpoint comparisons were non-significant (Figures 2G,G’; *p* = 0.1732).

Taken together, these findings suggest that synaptosomes derived from cryopreserved tissues retain the capacity for depolarisation-evoked Ca^2+^ entry, which scales with increasing extracellular potassium ([K^+^]_o_) and is abolished in the absence of extracellular Ca^2+^ or after synaptosomal denaturation. The inability of CdCl₂ to fully suppress the KCl-evoked response, together with the lack of additive effects from combining selective VGCC blockers, suggested that not all of the KCl-evoked response is explained by the VGCC pathways tested here. Although mean Ca^2+^ responses were comparable between fresh and frozen preparations, increased variability in cryopreserved samples suggests that freeze–thawing may perturb presynaptic membrane organisation or VGCC coupling.

**Figure 3:**
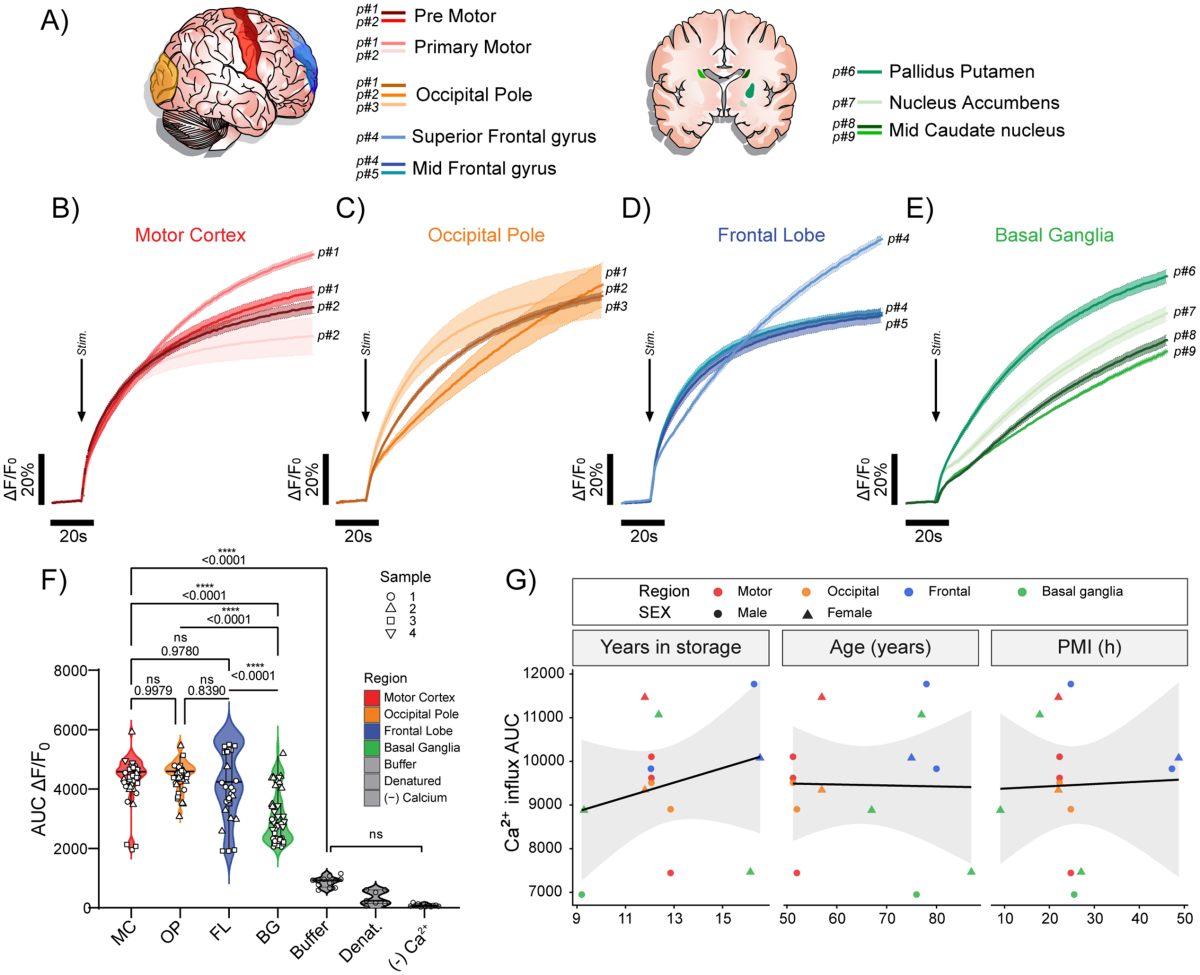
KCl-evoked Ca^2+^ mobilization in synaptosomes isolated from distinct human brain regions. **(A)** Anatomical schematics indicating sampled cortical and subcortical regions. **(B–E)** Post-mortem human synaptosomes from motor cortex (red gradient), occipital pole (orange gradient), frontal lobe (blue gradient), and sub-cortical structures including the basal ganglia (green gradient; mid-caudate, pallidum, and putamen) were depolarized with 30 mM KCl (arrow). Traces show mean (solid line) ± SD (shaded bounds) of ΔF/F₀ (% of baseline, initial 10–20 s) normalized to synaptosomal protein (μg) from 6 to 9 technical replicates per region and patient. **(F)** Ca^2+^ mobilization quantified by area-under-the-curve (AUC). Regional AUC distributions, including the Ca^2+^-free KCl and denatured controls (grey), are shown as violin/boxplots (violin: distribution; box: median and IQR). AUC varied across regions with substantial within-region variability, with the largest differences observed between cortical and subcortical structures. Violin/boxplots show distribution (violin), median and IQR (box); overlaid points are subject means. **(G)** AUC was not significantly associated with specimen covariates, including storage duration at −80 °C, donor age, or post-mortem interval (PMI). Points represent donor means colored by region (as in **A**); lines indicate least-squares regression with 95% confidence intervals.

We next examined K^+^ depolarisation-evoked Ca^2+^ mobilisation in human synaptosomes and quantified Ca^2+^ mobilisation from anatomically defined regions (Figure 3A). Depolarisation evoked clear ΔF/F_0_ increases in synaptosomes from all regions, with evident differences in response amplitude and kinetics across regions (Figures 3B–E). Quantification of the integrated response (AUC of ΔF/F₀) showed regional variation, with the strongest bulk responses observed in cortical regions and comparatively lower responses in basal ganglia, alongside substantial within-region variability consistent with heterogeneous synaptosome-enriched populations (Figure 3F). No difference was observed between motor and occipital cortex (Figures 3B,C). Negative controls (grey violin plots) showed minimal responses, confirming that the measured signal reflects stimulus-evoked Ca^2+^ entry (Figure 3F). To determine whether this variability resulted from patient-specific factors or differences in autopsy material quality, we examined correlations between Ca^2+^ influx measurements and patient demographics, including years in storage (YIS), age, and post-mortem interval (PMI) (Figure 3G). Pearson’s correlation tests revealed no significant associations between these factors and synaptosomal Ca^2+^ influx (Figure 3G). Collectively, these data indicate that while depolarisation drives Ca^2+^ influx across the human forebrain, the magnitude of the bulk synaptosomal response is region dependent and varies substantially between each region.

### Synaptosomes isolated from MND-affected motor cortices and hSOD1^G93A^ mice show increased depolarization-evoked Ca^2+^ influx

**Figure 4:**
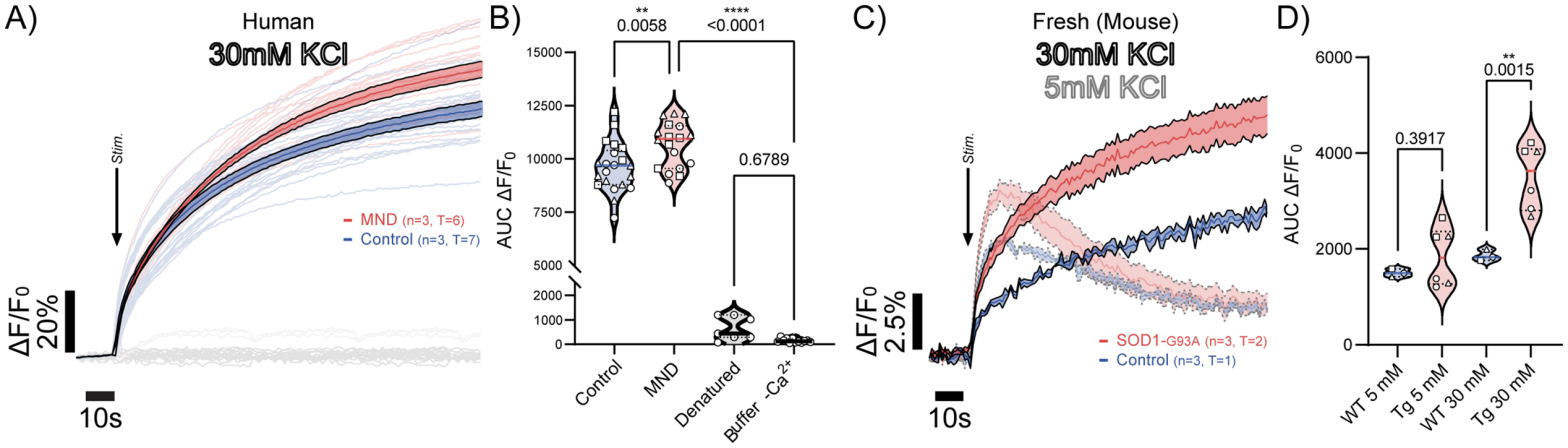
Increased depolarization-evoked Ca^2+^ mobilization in synaptosomes from human MND motor cortex and hSOD1^G93A^ transgenic (Tg) mice. **(A)** Post-mortem synaptosomes isolated from motor cortex of MND donors (red) or neurologically normal controls (blue) were depolarized with 30 mM KCl (arrow “Stim”). Individual replicate traces (opaque lines) and mean responses ± SEM (solid lines) are shown (*n* = 3 cases per group, 6–7 technical replicates per case). Black, grey, and light grey traces represent buffer-only, denatured sample, and Ca^2+^-free buffer controls, respectively. **(B)** Quantification of Ca^2+^ mobilization by AUC analysis of ΔF/F₀ revealed significantly increased Ca^2+^ responses in MND motor cortex compared to controls in this cohort (**p = 0.0058), with both groups showing greater responses than buffer and denatured controls (****p < 0.0001; one-way ANOVA with multiple comparisons). **(C,D)** Synaptosomes prepared fresh from whole cortex of control (blue) and hSOD1^G93A^ (red) mice were depolarized (“Stim” arrow) with 5 mM (opaque lines) or 30 mM KCl (solid lines). Thick lines show mean ΔF/F₀ ± SEM (*n* = 3 mice per group, 6–7 wells per mouse). AUC quantification demonstrates elevated KCl-evoked Ca^2+^ mobilization in hSOD1^G93A^ synaptosomes at 30 mM KCl compared to wild-type (WT) (**p = 0.0015; two-way ANOVA with multiple comparisons).

Having established that cryopreserved human synaptosomes retain ultrastructural integrity and functional responsiveness to depolarisation, we next examined whether these preparations could be used to interrogate disease-associated alterations in Ca^2+^ handling. Synaptosomes were isolated from motor cortices of neuropathologically defined control and MND donors. Upon high K^+^ depolarisation, synaptosomes from MND motor cortices showed greater depolarisation-evoked Ca^2+^ influx compared to control preparations, as evident from both the time course traces (Figure 4A) and quantification of AUC peak responses (Figure 4B). This enhancement was consistent across our donor cohort and showed no association with post-mortem interval or years in storage, suggesting that the observed responses are unlikely to be driven by storage-related artefacts.

To determine whether elevated Ca^2+^ influx is conserved across models, we next prepared synaptosomes from the whole cortex of hSOD1^G93A^ mice, a well-established genetic model of MND. Consistent with the human data, synaptosomes from hSOD1^G93A^ cortices exhibited significantly greater depolarisation-evoked Ca^2+^ entry relative to wild-type controls (Figures 4C,D). The increased Ca^2+^ influx was reproducible across independent preparations and comparable in magnitude to that observed in human MND motor cortex.

### Reduced complex IV-linked respiration in synaptosome-enriched fractions from MND motor cortex

**Figure 5:**
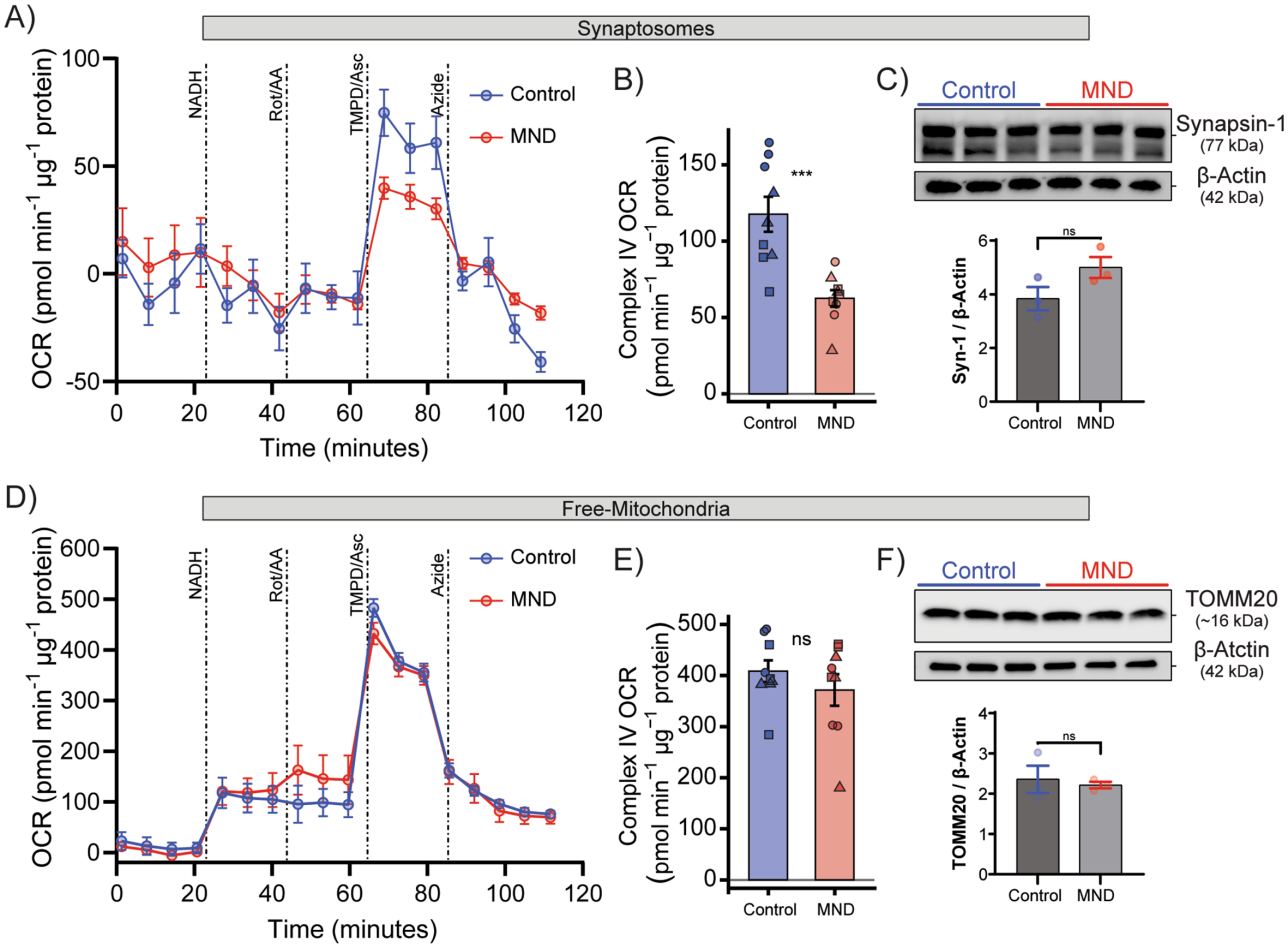
Selective impairment of Complex IV–driven respiration in synaptosome-enriched fractions from MND motor cortex with preserved free-mitochondrial content. **(A)** Oxygen consumption rate (OCR; pmol O₂/min, normalised per μg protein per well) of synaptosomes isolated from cryopreserved human motor cortex of control (blue, *n* = 3) and MND (red, *n* = 3) donors. Sequential additions of NADH (1 mM), Rotenone/Antimycin A (ROT/AA; 2 μM), TMPD (1 mM) plus Ascorbic acid (0.5 mM), and Na^+^ Azide (50 mM) were delivered via Seahorse XFe96 injection ports (vertical dotted lines). **(B)** Complex IV OCR (pmol O₂/min, normalised per μg protein per well), calculated as TMPD/Ascorbate-stimulated OCR minus the Azide-sensitive OCR fraction. **(C)** Western blot analysis of synaptosomal fractions probed for Synapsin-1 (~77 kDa) with *β*-Actin (~42 kDa) as a loading control. Quantification confirms comparable synaptic enrichment between control and MND samples. **(D)** OCR (pmol O₂/min, normalised per μg protein per well) of free mitochondria isolated from the same motor cortex donors and assayed under identical ETC challenge conditions. Sequential additions of NADH, Rotenone/Antimycin A, TMPD plus Ascorbic acid, and Na^+^ Azide were delivered via Seahorse XFe96 injection ports. **(E)** Free-mitochondrial Complex IV OCR (pmol O₂/min, normalised per μg protein per well), showing no significant difference between control and MND donors. **(F)** Western blot analysis of mitochondrial fractions probed for TOMM20 (~16 kDa) with β-Actin (~42 kDa) as a loading control, confirming comparable mitochondrial content between groups. Data are presented as mean ± SEM of six technical replicates per condition for Seahorse experiments, derived from three independent donors per group. Statistical comparisons were performed using unpaired two-tailed Welch’s *t*-tests.

Given current evidence for mitochondrial impairment in MND, we next assessed oxygen consumption in synaptosomes isolated from cryopreserved human motor cortex of control and MND donors using a sequential ETC challenge protocol (NADH → Rotenone/Antimycin A (Rot/AA) → TMPD/Ascorbate → Na^+^ azide), similar to assays previously validated in frozen tissues (Acin-Perez et al., 2020) (Figure 5). ECAR was recorded but is not interpreted here because the permeabilised assay conditions preclude robust attribution to glycolytic flux. NADH injection showed no difference between synaptosomal OCR in control and MND (Figure 5A; injection 1). ROT/AA inhibitory activity on OCR was similar between control and MND groups (Figure 5A; injection 2). Driving Complex IV with TMPD/Ascorbate produced a robust rise in synaptosomal OCR in all donors, but the peak response was lower in MND than in controls (Figure 5A; injection 3). TMPD/Ascorbate-driven respiration was subsequently inhibited by the Complex IV inhibitor Na^+^ azide, supporting the interpretation that the measured oxygen consumption reflects a mitochondrial, Complex IV-linked component (Figure 5A; injection 4). Complex IV–driven OCR was significantly reduced in MND synaptosomes relative to neurotypical controls, as indicated by diminished TMPD/Ascorbate-stimulated respiration and a smaller Azide-sensitive fraction (OCR_TMPD_-OCR_Azide_; Figure 5B). Synaptosome content in OCR respirometry experiments did not differ significantly between control and MND tissues, as assessed by synapsin-1 levels in paired donor-derived fractions (Figure 5C).

To determine whether the Complex IV effect observed in synaptosomes from control and MND donors is specific to the synaptosome-enriched fraction, we isolated donor-matched free-mitochondrial fractions from the same motor cortex samples and assessed OCR in mitochondrial assay solution (MAS) (Figure 5D). Because these preparations are cell-free, ECAR was not assessed. Following baseline stabilisation, addition of NADH increased OCR, indicating that frozen–thawed, permeabilised mitochondrial preparations retained redox-responsive oxygen consumption under these conditions. This response was largely insensitive to Rotenone/Antimycin A, suggesting that it was not mediated via the canonical Complex I–III pathway. By contrast, TMPD/ascorbate robustly stimulated OCR that was abolished by Na^+^ azide in both groups, confirming a mitochondria-linked, Complex IV–dependent component (Figure 5D). Complex IV–driven OCR did not differ significantly between MND and control mitochondria (Figure 5E), despite equivalent mitochondrial protein loading, as indicated by TOMM20 abundance (Figure 5F). The absence of a Complex IV OCR deficit in free-mitochondrial assays, contrasted with selective attenuation in synaptosomes from control and MND human brain, suggests that synaptosome-enriched preparations may provide greater sensitivity for detecting nerve terminal–associated bioenergetic alterations in this MND cohort. Collectively, these data show that cryopreserved synaptosomes retain partial ETC responsiveness, with a selective impairment observed in this MND cohort.

### Protein-load requirements and OCR scaling in synaptosomes and free-mitochondria from cryopreserved samples

From our bioenergetic assays on archived human brain, we quantified protein requirements for synaptosome and free-mitochondria preparations and defined the minimum amounts needed to quantify robust OCR responses. In synaptosomes, both baseline OCR and TMPD/Ascorbate–driven OCR showed a shallow, positive dependence on loaded protein abundance (Supplementary Figure S4A). By contrast, free-mitochondrial OCRs showed limited further increase with higher protein loading; empirically, ~65 μg per well was sufficient to achieve near-maximal Complex IV-driven OCR in isolated free-mitochondria (Supplementary Figure S4B). Synaptosome protein content correlated positively with MitoTracker signal, whereas the mitochondrial fraction showed no such correlation (Supplementary Figure S4C), likely reflecting sample-to-sample heterogeneity and dilution by non-mitochondrial proteins in the bulk free-mitochondrial preparation. To assess whether OCR measurements were influenced by donor-related variables, Pearson’s correlations were performed between bioenergetic parameters and donor age, years in storage, and post-mortem interval (PMI) (Supplementary Figure S4D). In synaptosomal preparations, no significant associations were observed between basal OCR or ΔOCR_TMPD/Asc_ and age, storage duration, or PMI, although a trend towards a negative correlation between PMI and basal OCR was noted (r = −0.75, *p* = 0.089). By contrast, free-mitochondrial preparations showed a significant negative correlation between PMI and basal OCR (r = −0.86, *p* = 0.026), while no significant associations were observed for ΔOCR_TMPD/Asc_. No significant relationships were observed between age or years in storage and OCR measurements in either preparation. Together, these findings indicate that while basal respiration may be modestly influenced by post-mortem interval, maximal Complex IV–driven respiratory capacity is largely preserved.

Overall, synaptosome-enriched fractions derived from cryopreserved human tissue yielded reproducible ultrastructural, Ca^2+^-handling, and bioenergetic readouts. Synaptosomes from MND motor cortex showed greater depolarisation-evoked Ca^2+^ entry and reduced Complex IV-driven respiration that was absent from donor-matched, free-mitochondrial fractions, suggesting that Complex IV impairment is detectable in the synaptosomes within this MND cohort. Together, these findings support cryopreserved human synaptosomes as an informative platform for detecting disease-relevant presynaptic phenotypes in archived brain tissue.

## Discussion

Cryopreservation and banking of human CNS tissue have largely been viewed as compatible with histology and multi-omics, but not with assays that depend on membrane integrity, Ca^2+^ entry, or respiratory-chain recruitment. Here we challenge that view. Using a workflow benchmarked in paired fresh and experimentally frozen mouse cortex and subsequently applied to cryopreserved human cortical tissue obtained at autopsy, we show that banked human tissue can yield synaptosomes that (i) retain the expected ultrastructure and synaptic proteome; (ii) display robust, pharmacologically tractable depolarisation-evoked Ca^2+^ entry; and (iii) support complex-specific OCR measurements sensitive enough to detect a disease-associated deficit in motor neuron disease (MND) motor cortex. Together, these data support that archived human brain tissue can support functional interrogation of presynaptic compartments and that synaptosomes provide a potentially more sensitive readout of MND-related bioenergetic change than bulk free-mitochondrial preparations.

### Feasibility and quality of synaptosomes from archived human tissue

Classic studies have shown that synaptosomes can be generated from previously frozen human and rodent brain; however, homogeneity, yield, and viability have not been rigorously benchmarked against fresh, low-PMI tissue using defined or matched cryopreservation protocols (Dunkley et al., 2008). Earlier human autopsy studies nonetheless demonstrated preserved morphology, linear O₂ uptake, K^+^/veratridine-evoked increases in respiration, and stimulus-evoked transmitter release, establishing feasibility for functional readouts in frozen human cortex (Nagy and Delgado-Escueta, 1984; Dodd et al., 1986; Hardy et al., 1986; Sherman et al., 1991). We address this by benchmarking synaptosomes from fresh and experimentally cryopreserved mouse cortex, with the latter prepared using the same isotonic sucrose protocol applied to human brain tissue archived for up to 26 years. Synaptosome ultrastructure, size, mitochondrial content and proteomics showed synaptosome fractions from cryopreserved human cortex enriched for pre- and post-synaptic elements with minimal nuclear contamination, consistent with signatures from freshly isolated rodent tissue (Evans, 2015). Depolarization evoked robust Ca^2+^ influx that varied across sampled regions and was not detectably associated with years in storage (≤26 y), donor age, or post-mortem interval.

Archived human synaptosomes were functionally excitable: K^+^- and veratridine-evoked ΔF/F₀ responses were robust and concentration-dependent, abolished by Ca^2+^ removal or denaturation, and partially sensitive to Na_V_ block (TTX), and to broad or subtype-selective VGCC inhibitors, consistent with partial preservation of Na_V_/VGCC-linked responses (Meder et al., 1999). Previous reports show K^+^-evoked Ca^2+^ influx is mediated predominantly by P/Q-type (*ω*-Agatoxin IVA–sensitive) channels, with a smaller contribution from N-type (ω-Conotoxin–sensitive) channels; L-type (Nifedipine-sensitive) channels do not measurably contribute under K^+^ depolarization (Meder et al., 1999). We observed only a modest, non-significant reduction with ω-Agatoxin IVA in our K^+^ paradigm, indicating that non-P/Q routes (e.g., N-type and a toxin-resistant component) may contribute to Ca^2+^ entry in our preparation. Notably, previous reports show human cortical synaptosomes treated with combined P/Q plus N, suppressed, but did not abolish K^+^-evoked Ca^2+^ signals (≈85% inhibition), and veratridine responses contained both a Nifedipine-sensitive fraction (L-type) and a small Na^+^/Ca^2+^ exchanger (NCX)-sensitive component (KB-R7943 ≈ 20% reduction), underscoring that incomplete pharmacological suppression is expected in mixed synaptosomal populations (Meder et al., 1999). Our observations on the incomplete suppression by Cd^2+^ or by P/Q-, N-, and L-type blockers are consistent with the interpretation that cryopreservation may alter channel coupling and/or unmask non-VGCC Ca^2+^ sources (e.g., mitochondrial release or Na^+^/Ca^2+^ exchange) in a subset of terminals (Nicholls and Budd, 2000; Rizzuto et al., 2012). Mouse synaptosome preparations have confirmed via electron microscopy the presence of broken membranes and underscored 30% of protein loading contributes to non-functional measurements (Choi et al., 2011). Consistent with these observations, veratridine (NaV) stimulation elicited only a modest, non-significant increase in OCR in our preparations, indicating that excitation-respiration coupling is attenuated following cryopreservation. This likely reflects the presence of unsealed membrane contaminants and other non-synaptic material, a well-recognised limitation of unsorted synaptosome fractions (Biesemann et al., 2014; Postupna et al., 2014). A key limitation of this approach is that, although synaptosome fractions are enriched for synaptic terminals with enclosed synaptic vesicles, they can contain myelin fragments, microsomes, and extra-synaptosomal mitochondria (Dunkley and Robinson, 2018), see Figure 1 and Supplementary Figure S3.

### Disease-linked exaggeration of Ca^2+^ entry in MND

In control cortices, we observed no differences in the magnitude or rate of depolarisation-evoked Ca^2+^ entry between occipital and motor regions. Synaptosomes derived from biobank tissues therefore provide a useful framework to investigate intra-donor Ca^2+^ handling, enabling direct comparison between affected motor regions and pathologically spared regions such as the occipital cortex (Brettschneider et al., 2013; Walhout et al., 2015; Machts et al., 2018). Future studies could also include additional stimulants such as glutamate to enhance physiological relevance (Battaglioli and Martin, 1990). Motor cortex synaptosomes from neuropathologically validated MND donors, as well as synaptosomes from hSOD1^G93A^ fresh mouse cortex assayed under identical conditions, exhibited exaggerated depolarisation-evoked cytosolic Ca^2+^ influx versus their respective controls. These findings converge with evidence for cortical hyperexcitability, impaired Ca^2+^ buffering, altered presynaptic signaling in ALS/MND (Kruman II et al., 1999; Vucic et al., 2008; Grosskreutz et al., 2010), and with reports of heightened Ca^2+^ transients and excitotoxic vulnerability in human iPSC-derived models (Wainger et al., 2014; Burley et al., 2022). Prior work further suggests that mutant hSOD1-driven presynaptic Ca^2+^ dysregulation is not solely due to enhanced active-zone VGCC opening, but may instead reflect abnormal release and impaired buffering of Ca^2+^ from intracellular stores, including mitochondria (Song, 2020). Nonetheless, the Ca^2+^ phenotype is detectable after years of cryostorage, compared to freshly prepared hSOD1^G93A^ mouse synaptosomes, supports its biological relevance rather than a storage artefact.

### Synapse-restricted Complex IV deficit with preserved free-mitochondrial respiration

Using synaptosomes and a sequential ETC challenge, both control and MND groups retained stimulus–response coupling, but MND synaptosomes showed a selective attenuation at Complex IV (reduced TMPD/Ascorbate-evoked, Azide-sensitive OCR). In free-mitochondrial fractions isolated from the same donors, no MND–control differences were detected. This divergence is consistent with a synaptosome-specific bioenergetic deficit; namely, a property of nerve terminals and their attached mitochondria, rather than a global failure across all mitochondria in end-stage tissue. The pattern is consistent with distal ETC vulnerability and Complex IV dysfunction described in ALS-affected regions (Mattiazzi et al., 2002; Wang et al., 2016), and is technically enabled by frozen-sample bioenergetic protocols that support complex-specific interrogation (Acin-Perez et al., 2020). Converging evidence shows frequent Complex IV abnormalities in sporadic ALS, including ~46% Complex IV-negative fibers in muscle biopsies and ~50% of cases with low-level truncating Complex IV mtDNA variants in blood (Crugnola et al., 2010; Baxi et al., 2022; Cheng et al., 2025). In rats, neuronal Complex IV deficiency is sufficient to drive ALS-like pathology, including selective *α*-motor-neuron loss, neuromuscular junction denervation, TDP-43 mis localization and progressive paralysis (Cheng et al., 2025). These observations are consistent with the interpretation that the reduced Complex IV-linked OCR observed in our synaptosome-enriched fractions reflects a disease-relevant compartmental vulnerability.

Several limitations should be considered when interpreting these findings. First, the human functional studies were necessarily performed on small cohorts of archived tissue due to tissue availability, which limited statistical power and precluded meaningful sex-stratified analyses (McCombe and Henderson, 2010). In addition, only male hSOD1^G93A^ mice were examined to maintain cohort consistency and reduce biological variability, as sex is recognized as a modifier of disease onset and progression in this model (Gurney et al., 1994; Veldink et al., 2003; Heiman-Patterson et al., 2005). Second, these synaptosome preparations are enriched rather than pure and likely contain a mixture of resealed terminals, extra-synaptosomal mitochondria, myelin fragments, and partially disrupted membranes. Cryopreservation and fractionation may further alter membrane integrity, channel coupling, and excitation–respiration relationships. Accordingly, the functional measurements reported here should be interpreted as arising from a synapse-enriched subcellular fraction rather than a homogeneous population of intact presynaptic terminals. This interpretation is supported, in part, by our mass spectrometry–based proteomic analysis of this subcellular fraction, which shows unbiased confirmation that this isolated fraction was enriched for synaptic proteins and pathways, thereby supporting our functional studies and conclusions.

Finally, the Seahorse assays were performed in permeabilised preparations and are therefore best interpreted as measures of respiratory-chain capacity, particularly Complex IV activity, rather than intact physiological oxidative phosphorylation. Consistent with this, ECAR measurements and additional receptor-driven stimulation paradigms could not be robustly assessed in the current study; incorporating intact preparations and appropriate metabolic controls (± glucose and ± 2-deoxyglucose) will be required to validate ECAR as a measure of glycolytic flux in this system. Despite these constraints, the concordance of ultrastructural, proteomic, biochemical, and functional readouts supports the biological relevance of the phenotypes identified in cryopreserved human tissue. Together, these data support the feasibility of using archived human CNS tissue to generate synaptosome-enriched fractions suitable for functional phenotyping, preserving depolarisation-evoked Ca^2+^ entry and enabling Complex-resolved respiratory measurements that reveal disease-linked changes. Integrated with multi-omic datasets, this approach may enable more scalable and mechanistic analysis of presynaptic function in archived human brain tissue.

## Data Availability

The original contributions presented in the study are included in the article/Supplementary material, further inquiries can be directed to the corresponding author/s.
