# Supplementary material for: Synaptosomes isolated from cryopreserved MND motor cortex reveal altered calcium handling and reduced complex IV–linked respiration
Source: Front Synaptic Neurosci. 2026 May 22;18:1760254. doi: 10.3389/fnsyn.2026.1760254 (PMC13236659; doi:10.3389/fnsyn.2026.1760254)
Supplement: Supplementary file 2 [file Supplementary_File_1.docx]

Supplementary Material

**Supplementary Methods.**

**Transmission Electron Microscopy:** Individual batches of synaptosomes (mouse frozen, fresh, and human frozen control) were prepared for electron microscopic analyses. Synaptosome pellets were immersed in 2.5% glutaraldehyde in 0.1 M Sodium Cacodylate buffer pH 7.4, overnight at 4°C. The pellets were then washed in 0.1 M Sodium Cacodylate buffer and post-fixed in 1% Osmium Tetroxide in 0.1 M Sodium Cacodylate buffer for 1 hr at room temperature. The synaptosomes were then rinsed in water and dehydrated through a graded series of ethanol solutions (30%, 50%, 70%, 100%) and twice in 100% acetone. Samples were infiltrated with epoxy resin, then embedded and polymerized at 65°C for 12‒24hrs. Ultra-thin sections (~200 nm) were cut from the resin blocks with a Leica EM UC6 ultramicrotome (Leica Microsystems, Sydney, Australia) and stained with Uranyl Acetate (5% in 50% ethanol) and 3% Lead Citrate to enhance contrast. Electron microscopic images of synaptosomes were captured at x12K magnification using a JEM-1011 electron microscope, operated at 80kV (Jeol, Pleasanton, CA, USA). These electron micrographs were then processed using ImageJ/FIJI to quantify organelle size (Schindelin, Arganda-Carreras et al. 2012). Selected EM micrographs were processed for contrast and brightness using Adobe Photoshop (Adobe, USA).

**Proteomic identification of postmortem synaptosomes - sample digestion:** Synaptosomes prepared from fresh and frozen WT-mouse brain, human-control (non-MND), and MND disease tissue were prepared for proteomics. Synaptosomes were lysed in RIPA buffer (150 mM NaCl, 1.0% IGEPAL® CA-630, 0.5% Sodium Deoxycholate, 0.1% SDS, 50 mM Tris, pH 8.0) and centrifuged for 10 minutes at 3000g, 4°C to remove cellular debris. The supernatant protein concentration was determined via PierceTM BCA Protein Assay Kit (ThermoFisher scientific, USA). The product was centrifuged in a 10 kDa molecular weight membrane column (Merck (Amicon®, USA) Ultra-0.5) to remove contaminating lipids and detergents ahead of digestion and subsequent LCMS, using a filter-aided sample preparation (**FASP**) protocol as described by Wiśniewski and colleagues (Wiśniewski, Zougman et al. 2009). A wash solution consisting of 8 M Urea and 50 mM Ammonium Bicarbonate (**ABC**) was applied (500 µl) to the filter column membrane and centrifuged for 14000 x g at room temperature and repeated 3 times. Wash solution plus 5 mM dithiothreitol was added to the filter column and incubated for 56˚C for 30 minutes. After incubation, the solution was brought to room temperature, and Iodoacetmide (**IAA**) was added at a final concentration of 25 mM. The alkylation reaction was performed in the dark for 30 minutes at room temperature. Dithiothreitol (**DTT**; 1 µl of 1 M stock) was added to quench IAA, and the filter column was spun at 14000 x g at room temperature until the solution passed the membrane. The filtrate was discarded, and 50 mM ABC was added, followed by Trypsin in a 1:50 trypsin: protein ratio (μg/μg). The filter column was incubated at 37˚C overnight on a shaker. The subsequent fractionate was equipped with a new collection tube, and centrifuged at 14000 x g until the solution passes the membrane. A subsequent centrifugation step with 0.5 M (50 µl) of NaCl was added to filter remaining peptides. The fractionate was prepared for a standard C18 Ziptip clean-up (Merck Sigma Aldrich - USA), as per the manufacturer’s instructions and concentrated by Speed Vac (Fischer Scientific) operating at 40°C (8 mbar, -OH) for 10 minutes. The evaporated product was suspended in 0.1% formic acid (20 µl) and transferred to Agilent glass MS vials for protein identification via Liquid Chromatography with tandem mass spectrometry (**LC-MS-MS**).

**Synaptosome proteomics:** Synaptosome proteomics was performed by LC–MS/MS using a Shimadzu Prominence nanoLC system coupled to a TripleTOF 5600 mass spectrometer (AB SCIEX, Framingham, MA, USA), as previously described (e.g., (Yeo, Chrysanthopoulos et al. 2016)). Strong cation exchange (**SCX**)-separated fractions (1–8; ~1 µg) and unfractionated samples (~1 µg) were loaded onto the autosampler maintained at 12 °C, trapped on a C18 column (150 µm ID × 150 mm, 5 µm particle size), and desalted for 3 min using 100% mobile phase at a flow rate of 30 µL/min. Peptides were separated on a Vydac Everest C18 analytical column (150 µm ID × 150 mm, 300 Å, 5 µm; Mandel Scientific, Canada) using a linear gradient of mobile phase AT (1% acetonitrile, 0.1% formic acid) and mobile phase BT (80% acetonitrile, 0.1% formic acid) at a flow rate of 1 µL/min. Injection volumes were 10 µL (~1 µg) for information-dependent acquisition (IDA) and 15 µL (~1 µg) for SWATH (Sequential Windowed Acquisition of all Theoretical fragment ions) experiments. Eluted peptides were analysed on the TripleTOF 5600 equipped with a Nanospray III ion source operating in positive ion mode using PicoTip emitter silica tips (12 cm, 10 µm diameter; New Objective, USA), with a de-clustering potential of 80 V, curtain gas of 30 psi, Gas 1 of 10 psi, and an interface heater temperature of 150 °C. Protein identification was performed using ProteinPilot software v4.5 (AB SCIEX) from LC–MS/MS fragment spectra, with peptide sequences searched against the UniProt database (Mus musculus or Homo sapiens). Only proteins identified with ≥95% confidence were reported, and false discovery rate analysis was performed using the integrated software tools. FASTA headers were removed from identified proteins to obtain UniProt accession numbers, which were subsequently used for Gene Ontology enrichment analysis using GOrilla (Eden, Navon et al. 2009).

**References cited.**

Eden, E., R. Navon, I. Steinfeld, D. Lipson and Z. Yakhini (2009). "GOrilla: a tool for discovery and visualization of enriched GO terms in ranked gene lists." BMC Bionformatics **10**: 48.

Schindelin, J., I. Arganda-Carreras, E. Frise, V. Kaynig, M. Longair, T. Pietzsch, S. Preibisch, C. Rueden, S. Saalfeld, B. Schmid, J.-Y. Tinevez, D. J. White, V. Hartenstein, K. Eliceiri, P. Tomancak and A. Cardona (2012). "Fiji: an open-source platform for biological-image analysis." Nature Methods **9**(7): 676-682.

Wiśniewski, J. R., A. Zougman, N. Nagaraj and M. Mann (2009). "Universal sample preparation method for proteome analysis." Nat Methods. **6**(5): 359-362.

Yeo, K. Y. B., P. K. Chrysanthopoulos, A. S. Nouwens, E. Marcellin and B. L. Schulz (2016). "High-performance targeted mass spectrometry with precision data-independent acquisition reveals site-specific glycosylation macroheterogeneity." Anal Biochem. **510**: 106-113.

**Supplementary Table S1**

Supplementary Table 1: Clinical diagnosis, disease duration, and neuropathological findings in post-mortem motor neuron disease (MND) cases.

| **Case** | **Clinical Diagnosis** | **Duration (yr)** | **Neuropathological Features (MND)** | **Comorbid Pathology** |
| --- | --- | --- | --- | --- |
| **MND ^1^** | MND with frontotemporal dementia | 6 months | Motor neuron loss in anterior horns and brainstem nuclei; Bunina bodies; gliosis | Alzheimer-type pathology (Braak I–II), cortical plaques, microinfarcts |
| **MND ^2^** | MND | Several years (not specified) | Neuronal loss and gliosis in motor cortex, hypoglossal nucleus and anterior horn cells | Minimal diffuse plaques; no significant AD pathology |
| **MND ^3^** | MND | ~1 year | Betz cell loss; neuronal loss in cranial nerve nuclei and cervical anterior horns | Minor age-related Alzheimer-type changes in medial temporal lobe |
| **MND ^4^** | MND | Several years (not specified) | Neuronal loss in precentral gyrus and multiple brainstem motor nuclei | Alzheimer-type pathology (Braak I–II; CERAD 1) |
| **MND ^5^** | MND | ~8 years | Loss of large neurons in precentral gyrus; degeneration in dorsal vagal, hypoglossal and abducens nuclei; anterior horn involvement | Occasional neurofibrillary tangles; minimal AD-type pathology |
| **MND ^6^** | MND | ~1 year | Motor cortex neuronal loss with gliosis; degeneration of pyramidal tracts and cranial motor nuclei | Sparse ubiquitinated inclusions; no significant AD pathology |
| **MND ^7^** | MND | ~3 years | Loss of Betz cells and neuronal loss in precentral cortex; degeneration of cranial motor nuclei and pyramidal tracts | Mild hippocampal neurofibrillary tangles (age-related) |

**Supplementary Figure S1**

**
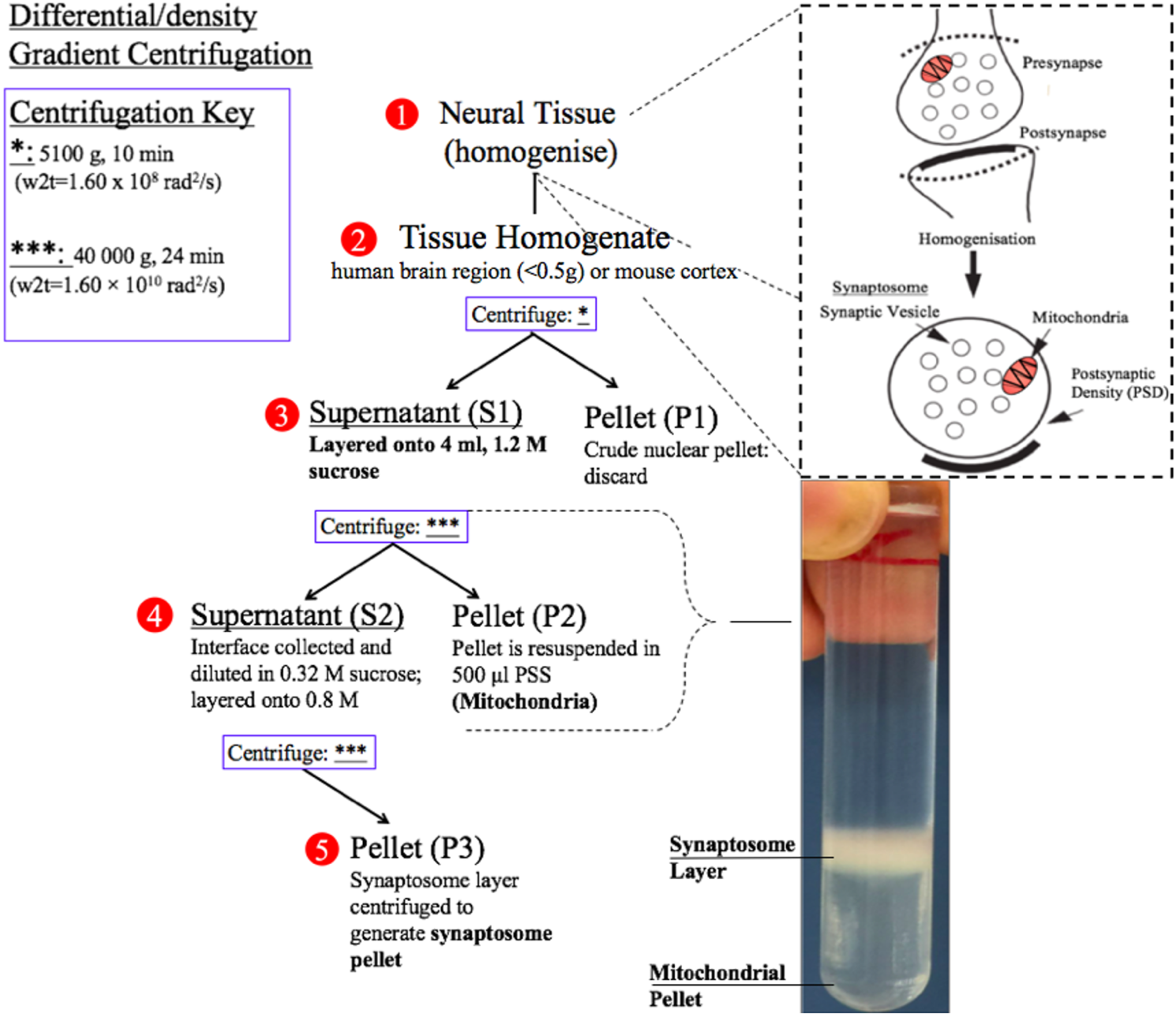
**

**Figure S1: Workflow for synaptosome isolation**. Synaptosomes were prepared using differential sucrose gradients on brain homogenates. Cortical tissue was removed from -80˚C storage, rapidly thawed in sucrose at 37˚C and held at 4˚C before homogenisation. Homogenisation was conducted in a Teflon glass homogeniser. The homogenate was centrifuged at a low speed; 1000-5000g is reported in the literature for 10 minutes. The S1 fraction was layered onto a 1.2 M (4 ml) sucrose step, and centrifuged at 40,000g for 16 minutes (total elapsed time 24 minutes include brake phase). The P2 pellet was retained for simultaneous analysis; this contained an enriched fraction of isolated mitochondria. The S2 interface was aspirated and diluted in 0.32M sucrose buffer. This was layered into 0.8M sucrose and centrifuged as per previous. The P3 pellet contained enriched mitochondria and was used for synaptosome analysis. All centrifugation was carried out in a Beckman Coulter ultracentrifuge with the SW41Ti rotor.

**Supplementary Figure S2**

**
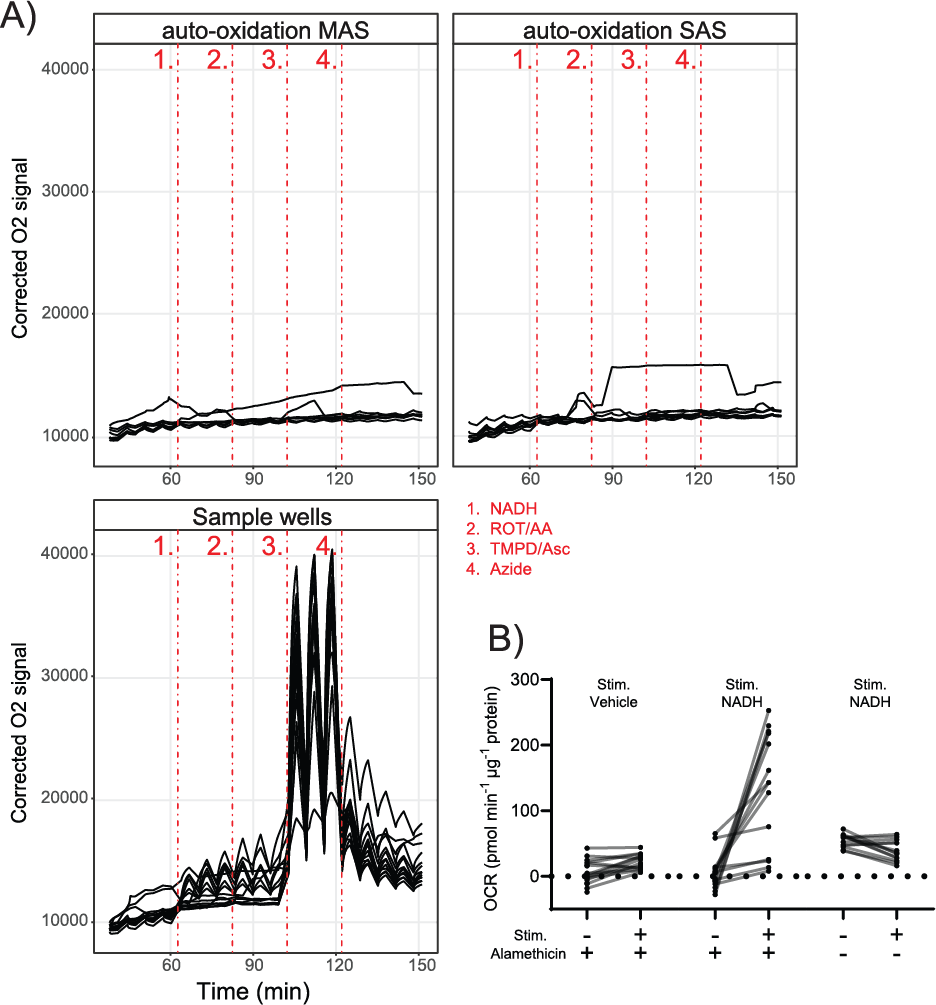
**

**Figure S2: Auto-oxidation controls and NADH-dependent stimulation of respiration. A**) Seahorse OCR traces showing control wells containing MAS or SAS without biological material and sample wells following sequential injections of NADH (1), ROT/AA (2), TMPD/Asc (3), and Azide (4). Control wells show no changes in O₂ signal, indicating negligible auto-oxidation. Sample wells display expected respiratory responses, with TMPD/Asc-driven maximal OCR abolished by azide. Red dashed lines indicate injection points. **B**) OCR (pmol O₂/min/µg protein) under NADH or vehicle stimulation ± Alamethicin. NADH alone does not increase OCR, whereas alamethicin permeabilisation enables a robust NADH-driven response, consistent with direct stimulation of respiration.

**Supplementary Figure S3**

**
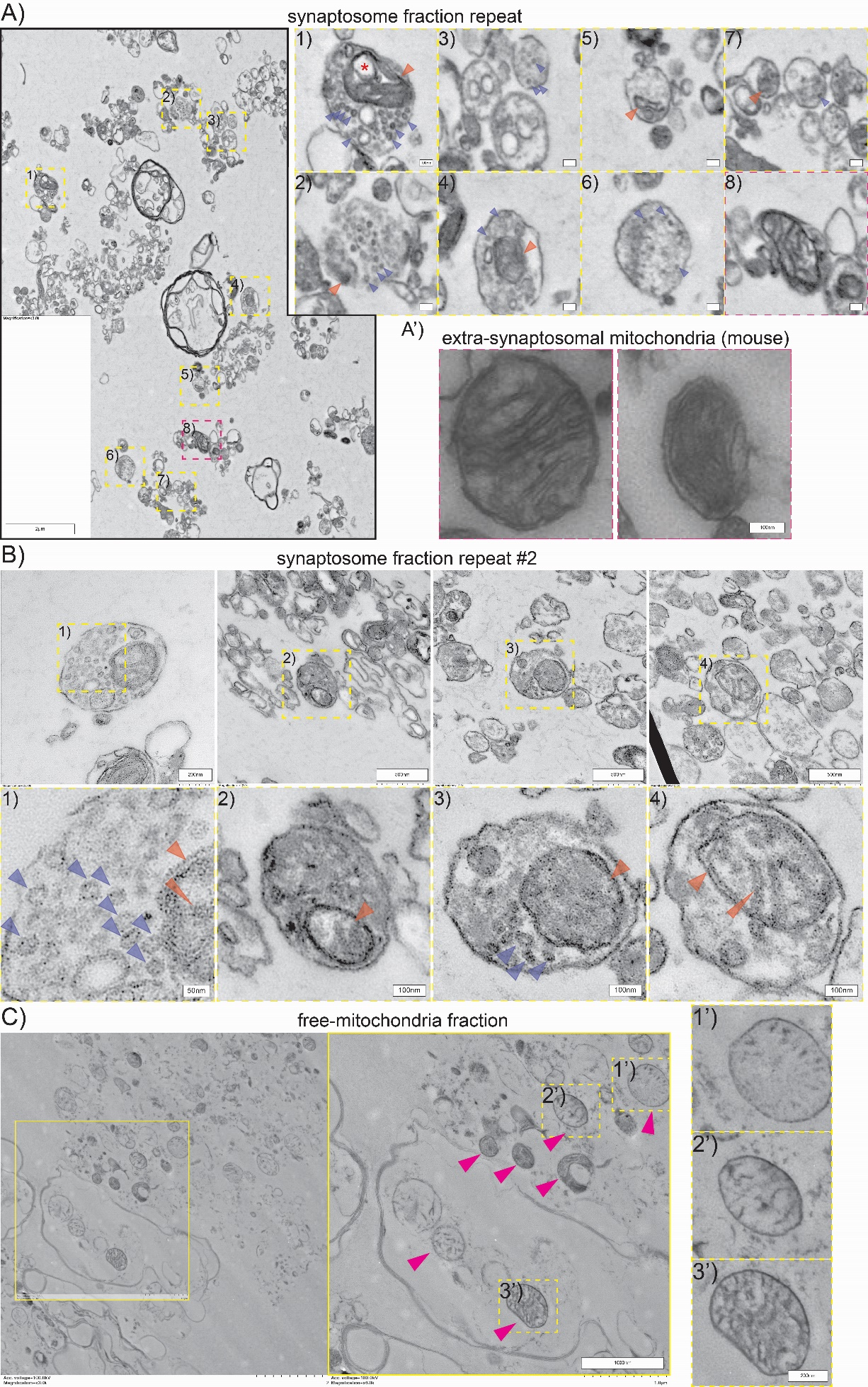
**

**Figure S3: Ultrastructural characterisation of synaptosome and mitochondrial fractions following freeze–thaw of human brain tissue. A**) Transmission electron microscopy (TEM) of synaptosome-enriched fractions demonstrating heterogeneous populations of resealed presynaptic terminals. Synaptic vesicles (20–40 nm; dark blue arrows) and intra-synaptosomal mitochondria (orange arrows) are evident. Mitochondria frequently display swollen morphology, consistent with freeze-thaw-associated structural alterations (red asterisk, inset 1). Scale bar = 2 µm; inset panels (1–8), 100 nm. **A’**) Representative images of extra-synaptosomal (free) mitochondria observed within synaptosome fractions, lacking surrounding presynaptic membrane structures. Regions highlighted with a pink border indicate contaminating mitochondrial profiles. Comparative images are included from freshly prepared mouse synaptosomes (same preparations as Figure 1B). **B**) Independent replicate TEM of synaptosome fractions derived from cryopreserved human tissue, confirming reproducibility of ultrastructural features. Synaptic vesicles are visible in panel B1, and mitochondria with intact double membranes and cristae are shown in panel B4. The image in panel B1 is duplicated and enhanced in main text; Figure 1C. Scale bars as indicated. **C**) TEM of the free mitochondrial fraction, showing enrichment of mitochondria devoid of enclosing synaptosomal membranes; pink arrows. Scale bar = 1 µm; inset panels (1′–3′), 200 nm.

**Supplementary Figure 4**


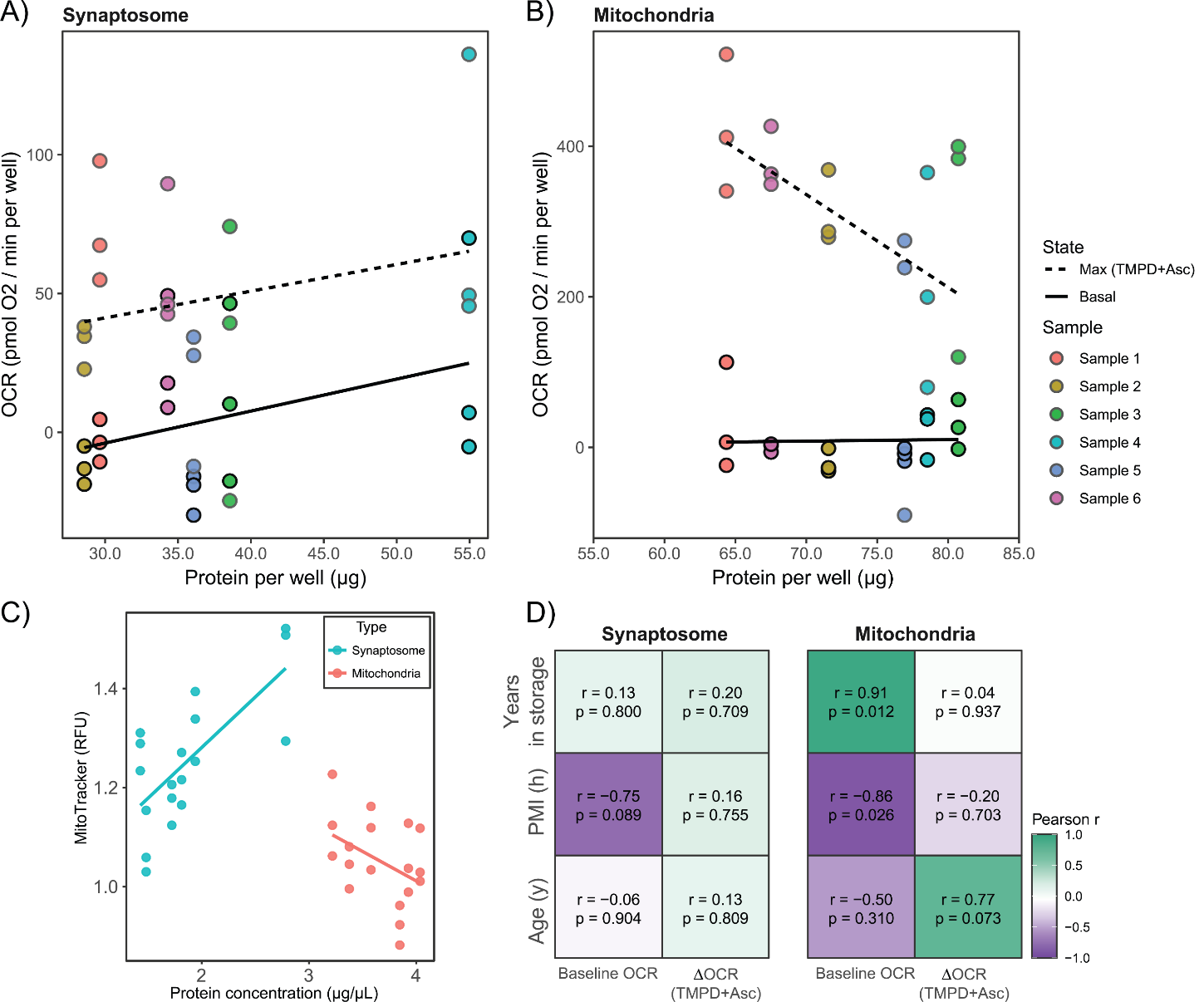


**Figure S4: Protein load and oxygen consumption rate (OCR) responses in synaptosome and mitochondrial fractions. A–B**) Basal and maximal OCR (TMPD/ascorbate-driven) plotted against loaded protein amounts for synaptosomes (**A**) and mitochondria (**B**) prepared from six independent human brain samples. **C**) Relationship between protein concentration (µg/µL) and raw MitoTracker fluorescence, showing a positive association in synaptosome fractions and a negative association in mitochondrial fractions. **D**) Correlation matrices of basal and maximal OCR with years in storage, post-mortem interval (PMI), and donor age. A negative association was observed between PMI and basal OCR in synaptosomes, which was absent in maximal, ΔOCR_TMPD+Asc_ measures.
